# Spatiotemporal feature-based investigation of major depressive disorder and emotion recognition using functional near infrared spectroscopy

**DOI:** 10.3389/fpsyt.2026.1907020

**Published:** 2026-10-06

**Authors:** Jinlong Chao, Rui Yang, Hong Peng, Jian Shi, Qiuxia Shi

**Affiliations:** 1 School of Computer & Artifical Intelligence (College Of Software), Lanzhou University of Technology, Lanzhou, China; 2 Guangyuan Mental Health Center, GuangYuan, China; 3 School of Information & Engineering, Lanzhou University, Lanzhou, China

**Keywords:** deep learning, emotion recognition, functional near-infrared spectroscopy (fNIRS), major depressive disorder(MDD), spatiotemporal feature

## Abstract

Major Depressive Disorder (MDD) remains one of the most prevalent psychiatric conditions worldwide, with a marked increase in incidence and severity following the COVID-19 pandemic. Despite its rising burden, clinical diagnosis continues to rely on subjective assessments, lacking objective and reproducible neurobiological markers. Functional Near-Infrared Spectroscopy (fNIRS) has emerged as a promising non-invasive imaging technique capable of capturing the spatiotemporal dynamics of cerebral hemodynamics, particularly within the prefrontal cortex—a key region involved in emotional regulation and cognitive control. In this study, we propose a spatiotemporal feature-based framework for MDD classification and emotion-related brain state recognition. Thirty MDD patients and thirty demographically matched healthy controls underwent fNIRS recording while exposed to emotional auditory stimuli. By analyzing group-level differences in oxyhemoglobin concentrations and *β*_values derived from a general linear model (GLM), we identified significant prefrontal abnormalities in the MDD group, particularly under negative emotional conditions. These alterations were most prominent in the dorsolateral prefrontal cortex (DLPFC), a region consistently implicated in emotion dysregulation. The classification model, based on fNIRS-derived activation features, achieved 90% accuracy for MDD detection and 85% for emotion recognition. These results demonstrate that integrating spatial and temporal features improves sensitivity to depression-related cortical dysfunction, offering a potential objective biomarker for diagnosis and novel insight into emotion-specific neurocircuitry in MDD. This work supports the clinical feasibility of fNIRS-based biomarkers and advances understanding of affective brain processing in depression.

## 1 Introduction

Major Depressive Disorder (MDD) is a prevalent psychiatric condition primarily characterized by persistent low mood and cognitive impairment, with clinical manifestations including irritability, anhedonia, sleep disturbances, low self-esteem, and feelings of guilt; in severe cases, patients may exhibit recurrent suicidal ideation (1, 2). According to the World Health Organization, approximately 1 billion individuals across various age groups worldwide suffer from MDD. From 2015 to 2022, the global number of depression cases increased by 27.6%. However, fewer than half of those affected—and in some countries, fewer than 10%—receive accurate diagnoses and effective psychological treatment, primarily due to a lack of medical resources and trained clinical psychologists. Furthermore, research indicates that individuals with MDD often exhibit symptoms across behavioral, emotional, and cognitive domains, with a high risk of suicide and relapse (3). Statistics show that over 35% of patients experience multiple depressive episodes, typically relapsing within two years after the initial remission. If not diagnosed and treated in the early stages, MDD can have severe consequences for individuals, families, and society at large (4). In China, the diagnostic rate for MDD ranges from 3% to 6% (5), and recent years have seen an upward trend. Alarmingly, there is also an increasing tendency toward earlier onset of suicide cases related to depressive disorders.

Recent research has increasingly shown that excessive emotional suppression and the inability to express emotions effectively are key contributors to psychological disorders. Emotional expression involves complex, hierarchical information patterns, with network-level interactions spanning multiple brain regions (6). Affective disorders, primarily marked by mood disturbances, are linked to disruptions in cognition, behavior, psychophysiological responses, and interpersonal functioning. In Major Depressive Disorder (MDD), patients typically exhibit both functional and structural brain abnormalities, particularly impaired switching mechanisms within the limbic system (7). Neuroimaging studies have consistently revealed dysregulation in several brain regions in MDD patients, including the prefrontal cortex (PFC), amygdala, striatum, and thalamus (8).

Currently, one of the major challenges in psychiatric clinical practice is the lack of objective diagnostic markers. Existing diagnostic approaches for Major Depressive Disorder (MDD) heavily rely on patients’ verbal self-reports and assessments based on psychiatrists’ clinical experience and communication with the patient. As a result, the diagnostic and therapeutic processes remain highly subjective, with the accuracy of conclusions largely dependent on the physician’s expertise. Individual differences in clinical judgment and personal cognitive biases can significantly impact diagnostic outcomes (9). Furthermore, the diagnostic process for MDD often involves structured or semi-structured interviews, which are inherently intrusive and require patients to voluntarily disclose their mental states, including painful memories that they may be reluctant to revisit. Additionally, even among patients diagnosed with the same disorder, clinical symptoms often vary significantly, leading to high heterogeneity in presentation and limiting the generalizability of research findings across different patient populations. These challenges have made it difficult for current studies to identify reliable neuroimaging biomarkers for MDD within a single diagnostic framework. Therefore, identifying robust and reproducible neurobiological markers at the individual level could provide a critical foundation for understanding the disorder and enabling the implementation of precise, personalized medical interventions.

The brain is an energy-demanding organ, with neuronal activation closely linked to increases in cerebral blood flow and volume—an interaction known as “neurovascular coupling,” which underpins functional neuroimaging techniques. Functional near-infrared spectroscopy (fNIRS) does not directly measure neural activity but instead detects local changes in blood oxygenation, which are tightly associated with neuronal activity. This is similar to the principles of functional magnetic resonance imaging (fMRI). However, the physical principles for detecting blood oxygenation differ between the two methods: fNIRS relies on changes in the optical properties of brain tissue, while fMRI detects variations in local magnetic fields induced by blood oxygenation. With advances in data acquisition and analysis, fNIRS has become increasingly valuable for exploring neural mechanisms in cognitive processes, providing empirical support for cognitive science models. Recently, this technique has gained traction in clinical research, particularly in studying the pathophysiology and auxiliary diagnosis of psychiatric disorders (10–12).

Despite growing neuroimaging evidence, findings on functional brain alterations in individuals with Major Depressive Disorder (MDD) remain inconsistent across studies (13, 14). Some have reported reduced regional cerebral blood flow (rCBF) in the left frontal lobe, with reductions correlating positively with symptom severity, while others have identified diminished rCBF in the left cingulate cortex. Research using the Wisconsin Card Sorting Test (WCST) has shown hypoperfusion in the left frontal and temporal lobes at rest, which intensifies during task performance, with additional deficits observed in the right frontal lobe. Several factors may account for these discrepancies: (1) Variability in experimental paradigms and cognitive states—brain activity differs between rest and task conditions, and distinct tasks recruit different neural circuits; (2) Clinical heterogeneity—frontal perfusion may increase following pharmacological treatment, suggesting that metabolic changes may reflect transient state markers rather than stable traits. Differences are also observed between medicated and unmedicated individuals, those with or without suicidal behavior, and between first-episode and recurrent patients; (3) Differences in imaging modalities—functional techniques differ in underlying physical principles, as well as in spatial/temporal resolution, sensitivity, and specificity (15).

To date, numerous fNIRS studies have investigated the association between cerebral blood flow and the severity of depressive symptoms accompanied by anxiety (16–19). However, results remain inconsistent—some studies report significant positive correlations, while others find no meaningful differences. For example, Nishimura et al. examined patients with a history of panic attacks and found that oxygenated hemoglobin (HbO) changes in the left inferior prefrontal cortex were significantly associated with the frequency of panic episodes (20). Although verbal fluency task (VFT) performance did not differ significantly between groups, both suicidal subgroups showed lower average HbO concentrations than healthy controls, particularly in the bilateral orbitofrontal and dorsolateral prefrontal cortices. Beyond anxiety-related depression, numerous studies have investigated the association between cerebral blood flow (CBF), metabolic dysfunction, and the severity of depressive symptoms. However, findings remain inconclusive. For instance, Noda et al. reported a significant negative correlation between symptom severity and oxygenated hemoglobin (HbO) changes in the right dorsolateral prefrontal cortex (DLPFC) using near-infrared spectroscopy (NIRS) (21). In contrast, Kameyama et al. found no significant correlation between symptom severity and NIRS signals in patients with bipolar affective disorder (22). Collectively, these studies underscore the inconsistency of fNIRS findings in Major Depressive Disorder (MDD). Possible explanations include differences in imaging techniques, measurement sites, individual variability, and data processing protocols (15, 23).

In recent years, functional near-infrared spectroscopy (fNIRS) has been increasingly utilized for the identification of depressive disorders. For example, Zhu et al. employed a wearable fNIRS headband to monitor specific brain regions and extracted features such as the mean and kurtosis of HbO signals (24), achieving a classification accuracy of 92.6%. Ma et al.used fNIRS recordings during verbal fluency tasks in a cohort of 36 patients with Major Depressive Disorder (MDD) and 48 individuals with bipolar disorder (25). Their model, based on an attention-enhanced Long Short-Term Memory (ALSTM) network, achieved an accuracy of 96.2%. Although many studies have reported high classification performance, most rely on a single type of feature or classification strategy, limiting generalizability. Additionally, few have addressed emotion recognition in the context of depressive disorders. To bridge these gaps, the present study proposes a unified fNIRS-based framework that integrates both disorder classification and emotion recognition. By examining differences between MDD patients and healthy controls across neural activation patterns and emotional states, this work offers a more comprehensive approach for detecting depressive pathology and its associated affective signatures.

In summary, substantial progress has been made in understanding Major Depressive Disorder (MDD), with findings of both scientific and clinical significance (26–29). Nevertheless, fNIRS-based research on MDD continues to face methodological and practical challenges that warrant further refinement. (1) Limited methodological diversity and analytical depth-Current fNIRS studies on MDD largely rely on basic statistical comparisons of oxyhemoglobin (HbO) levels, often overlooking integrated analyses that combine hemodynamic signals with cortical activation patterns. Few investigations have bridged these dimensions, resulting in narrow methodological scope and superficial insight. Future research should adopt spatiotemporal integration strategies that merge temporal dynamics with spatial localization tomore accurately characterize MDD-related neural and emotional processes. (2) Inconsistent and insufficiently robust physiological biomarkers-Given the heterogeneity of MDD and the incomplete understanding of its neural underpinnings, a wide range of fNIRS-derived features—such as signal characteristics and functional connectivity—have been explored for diagnostic purposes (24, 25). However, extracting simple, reliable biomarkers from complex and noisy datasets remains a major challenge, limiting the clinical applicability of current models.

Most current studies rely on group-level analyses or supervised learning models that assume distinct biological differences between diagnostic groups and homogeneity within each group (24, 25). However, these assumptions are frequently invalidated by symptom overlap and misdiagnosis, leading to substantial within-group heterogeneity. This undermines classifier training and limits the generalizability of identified biomarkers. As fNIRS research advances, the number of extracted features has increased markedly, resulting in high-dimensional datasets. This complexity hampers the identification of clinically relevant information and the development of models based on a limited set of physiologically meaningful predictors. A key challenge is thus the development of efficient channel and feature selection strategies to extract robust, generalizable biomarkers for accurate and interpretable classification in depression and emotion recognition (30).

## 2 Materials and methods

### 2.1 Participants

This study included 60 participants: 30 individuals diagnosed with MDD and 30 healthy controls. MDD patients were recruited from the GuangYuan Mental Health Center between September 2020 and June 2022. All patients were evaluated by licensed psychiatrists using the Mini-Mental State Examination (MMSE), and demographic data were collected for statistical analysis. Symptom severity was assessed using the Patient Health Questionnaire-9 (PHQ-9) (31), Athens Insomnia Scale (AIS), and Generalized Anxiety Disorder Scale-7 (GAD-7) (32) to evaluate depression, sleep disturbances, and anxiety, respectively. Written informed consent was obtained from all participants. The study protocol was approved by the Ethics Committee of the Third People’s Hospital of Tianshui.

This work involved human subjects or animals in its research. Approval of all ethical and experimental procedures and protocols was granted by GuangYuan Mental Health Center Ethics Committee under Application No. GJWLSP2020012, and performed in line with the Ethical Review Measures for Biomedical Research Involving Human Subjects, Declaration of Helsinki, and International Ethical Guidelines for Biomedical Research Involving Human Subjects.

Descriptive statistics for participant demographics are shown in **Table 1**. No significant differences were found between the MDD and control groups in age (t = 1.031, p = 0.331), sex (*χ*^2^ = 2.301, p = 0.102), or years of education (t = 0.897, p = 0.478). In contrast, MDD participants scored significantly higher than healthy controls on the PHQ-9, PHQ-15, GAD-7, and AIS scales (*p* < 0.05).

**Table 1:** Demographic characteristics of MDD and HC groups.

Demographic and clinical characteristics	MDD	HC	*p*_Value
age	34.5 ± 9.1	36.8 ± 11.5	>0.05
sex	18 female/12 male	16 female/14 male	>0.05
education	9. 78 ± 3.19	9.72 ± 3.71	>0.05
PHQ-9	13.96 ± 6.31	3.45 ± 5.04	<0.001
PHQ-15	11.75 ± 4.96	4.61 ± 3.51	<0.001
GAD-7	11.49 ± 5.39	3.95 ± 4.67	<0.001
AIS	15.61 ± 4.74	5.74 ± 4.39	<0.001

Age, education, PHQ-9, PHQ-15, GAD-7, and AIS were analyzed using independent samples t-test; gender was analyzed using the chi-square test. Statistical significance was set at *p* < 0.05.

### 2.2 Experimental paradigm

An emotional audio stimulation paradigm was implemented, as illustrated in **Figure 1**. Audio clips were selected from a curated database and validated through pretests with multiple participants, confirming significant differences in valence and arousal across stimuli. Prior to data collection, participants received standardized instructions to ensure consistent understanding of the procedure. The experiment comprised two phases: a resting-state recording and a task-based recording. During the resting-state phase, participants were instructed to remain awake with their eyes closed for three minutes. A tone signaled the beginning and end of this phase, while a fixation cross was presented on the screen throughout the auditory stimulation period.

**Figure 1:**
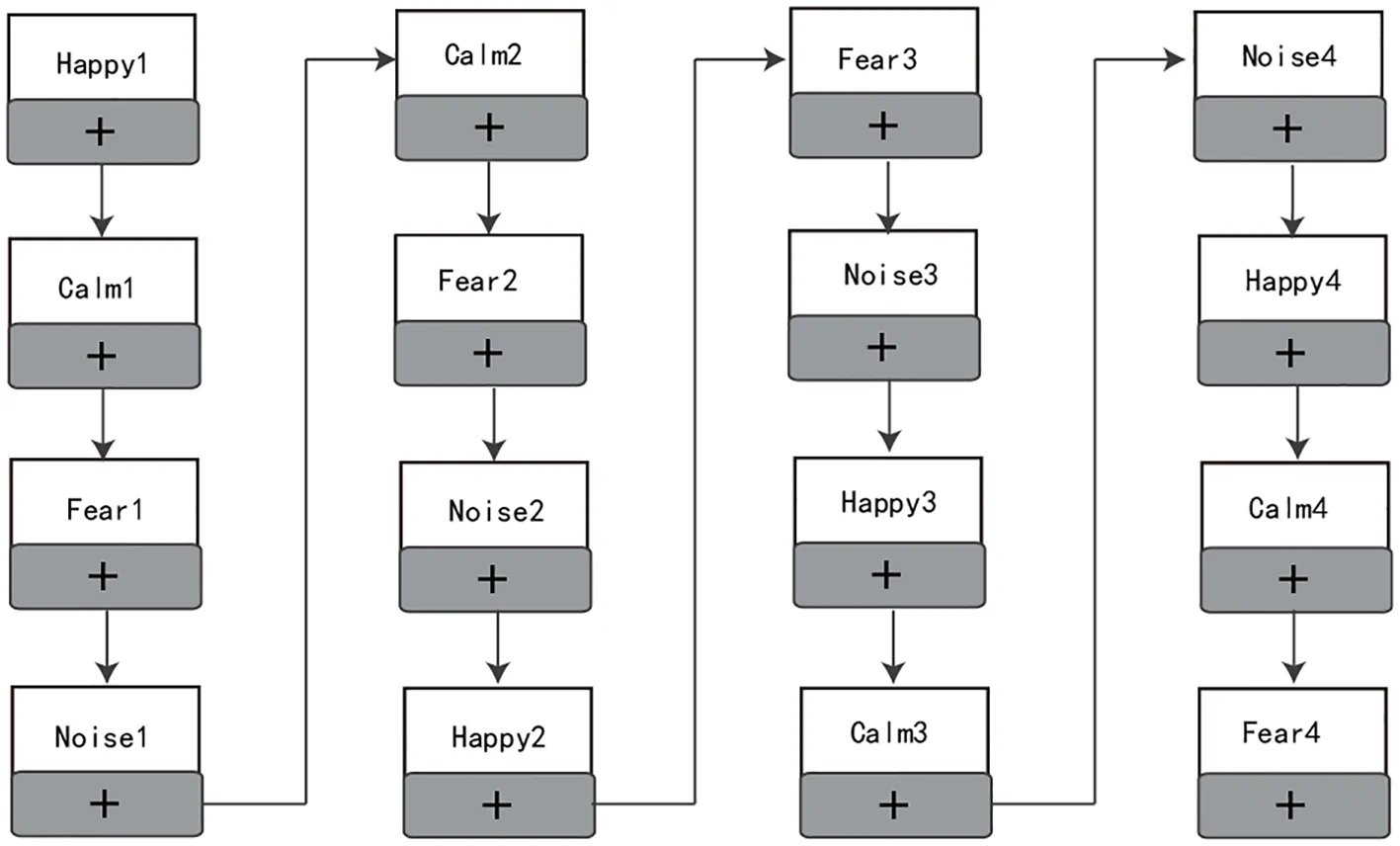
Emotion-inducing auditory paradigm.

In the task-based condition, the experiment consisted of 16 trials organized into four blocks, each containing four types of auditory stimuli: positive (happiness), neutral (calmness), negative (fear), and white noise. Stimuli were presented using a Latin square design to counterbalance order effects, a method well-suited for experiments with four or more conditions. Each stimulus lasted 18 seconds, followed by a 20-second intertrial interval to allow the hemodynamic response to return to baseline. The total duration of the task-based session was approximately 15 minutes. Participants were instructed to minimize body movement throughout the experiment to reduce motion-related artifacts in the fNIRS signals.

### 2.3 Channel configuration and data collection

**Figure 2** illustrates the optode configuration used in this study, where red and blue dots represent sources and detectors, respectively. The setup included 8 sources and 7 detectors, arranged with 3 cm spacing, forming 22 measurement channels (CH1–CH22) for recording cortical hemodynamic signals. These channels covered the left and right dorsolateral superior frontal gyrus, middle frontal gyrus, medial superior frontal gyrus, and portions of the orbitofrontal cortex.

**Figure 2:**
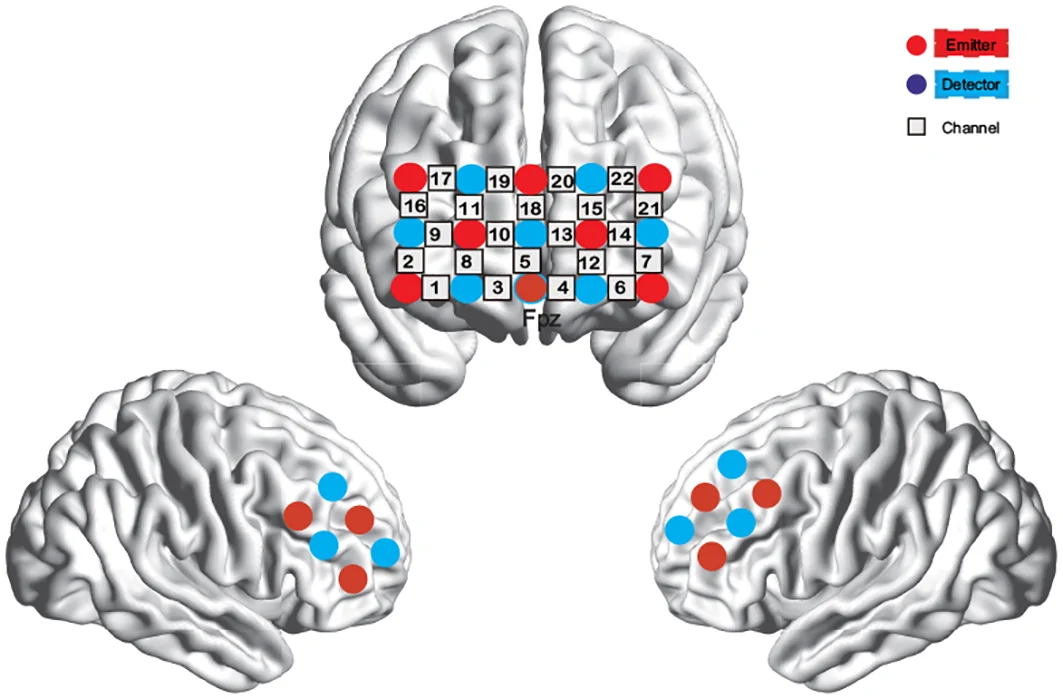
Layout of fNIRS channels over the prefrontal cortex.

The fNIRS system used in this study was the NIRScout desktop platform (NIRx, USA). This multi-channel system operates on continuous wave (CW) technology and measures relative concentrations of oxyhemoglobin (HbO), deoxyhemoglobin (HbR), and total hemoglobin (HbT). The measurement is based on the modified Beer–Lambert Law and the Matcher equation, which relate changes in optical density to hemoglobin concentration variations in cortical tissue.

Data acquisition was conducted in a quiet, light-shielded room. Participants wore an elastic headband (20 × 8 cm) to secure the optodes. At the start of the experiment, participants completed the task while cortical hemodynamic signals were simultaneously recorded. To ensure data quality, they were instructed to minimize physical movement, particularly head motion, to avoid optode displacement. Functional signals were recorded using NIRStar software (Version 15.1, NIRx) with dual wavelengths of 760 nmand 850 nm at a sampling rate of 7.81 Hz.

**Figure 3** presents the overall workflow of the proposed study. Participants first performed an emotion-inducing paradigm while fNIRS signals from the prefrontal cortex were recorded. After preprocessing, raw optical density signalswere transformed into HbO concentration changes. A GLM analysis was subsequently performed to estimate task-evoked cortical activation, from which β-values were extracted as activation features. These features were further subjected to channel selectionto identify the most discriminative brain regions for MDD classification. Finally, the selected features were input into machine learning classifiers to evaluate the effectiveness of the proposed framework in distinguishing MDD patients from healthy controls.

**Figure 3:**
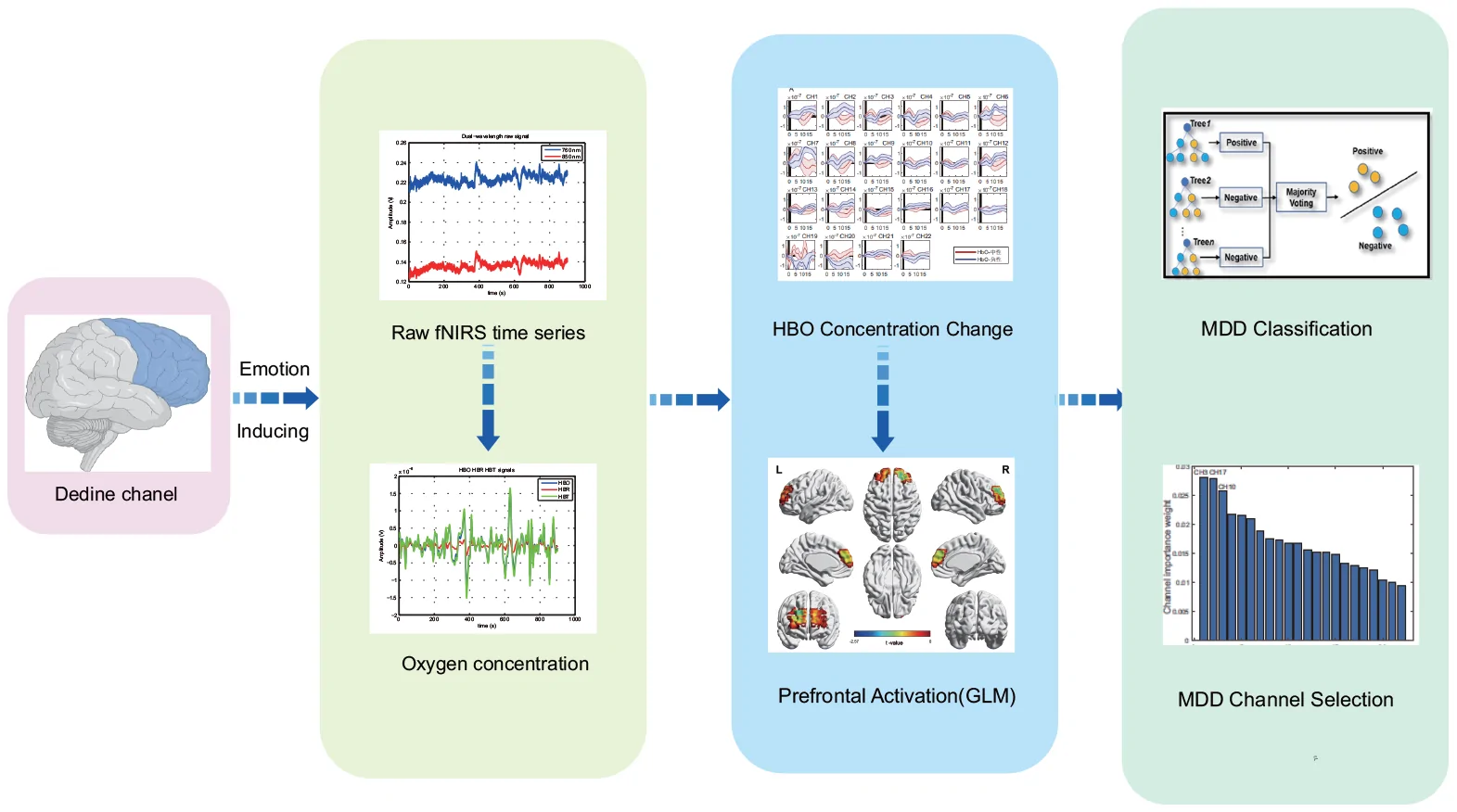
The overall workflow of the proposed study.

## 3 Group-level statistical analysis of hemodynamic concentration

### 3.1 Differences between MDD and healthy controls

Group-level analysis of hemoglobin concentration was conducted in three stages. First, for both the MDD and healthy control groups, mean oxyhemoglobin (HbO) concentrations were calculated across all 22 channels within a 5–20 second window following the onset of each emotional auditory stimulus. For each participant, channel-wise mean values were then averaged to obtain a single representative HbO value per condition. These values were subjected to statistical comparison between groups. Results of the group differences are summarized in **Table 2**.

**Table 2:** Statistical results of concentration differences between mdd and hc groups under different conditions (mean across 22 channels, unit: mol/L).

Result	Happy	Calm	Fear	Noise
MDD	0.18 ± 0.20	0.72 ± 0.24	0.59 ± 0.21	0.76 ± 0.21
HC	0.06 ± 0.11	0.43 ± 0.21	0.76 ± 0.18	0.73 ± 0.24
*t*_value	2.56^*^	3.49^**^	-3.53^**^	0.67

Values that are both bolded and underlined represent statistically significant differences ^*^p < 0.05, ^**^p < 0.01. Independent Samples t-test.

As shown in **Table 2**, significant group differences in oxyhemoglobin (HbO) concentration were observed under the happy, calm, and fearful auditory conditions, but not under the white noise condition. Accordingly, the white noise condition was excluded from further hemodynamic analyses. The corresponding t-values for the three significant conditions were 2.53^*^ 3.49^**^-3.53^**^, respectively.

MDD patients showed significantly higher mean HbO concentrations than healthy controls under the happy and calm conditions, but significantly lower concentrations under the fearful condition. This pattern suggests a distinct hemodynamic abnormality in response to negative emotional stimuli among individuals with MDD, characterized by reduced prefrontal oxygenation. These findings offer potential insights into the altered neural mechanisms of emotion processing in Major Depressive Disorder.

Channel-wise statistical analyses of mean oxyhemoglobin (HbO) concentrations (5–20 s window) were conducted for each emotional condition, and the results are presented in **Table 3**. Compared to the previous approach using a global average across all 22 channels, this analysis revealed more localized group differences. Specifically, significant differences between MDD and control participants were observed only under the fearful condition. CH3 showed a highly significant effect (p=0.002^**^ t=-3.198), while CH6 showed a moderate but significant difference (p=0.029^*^ t=-2.240). Notably, CH3 demonstrated the strongest effect, indicating a pronounced and region-specific hemodynamic abnormality in response to fear-related auditory stimuli.

**Table 3:** Group comparisons of mean HbO concentration (5–20 s) under different emotional stimuli.

Channel	Positive		Neutral		Negative	
	*p*	*t*	*p*	*t*	*p*	*t*
CH3	0.412	0.826	0.546	0.608	0.002^**^	-3.198
CH6	0.106	1.641	0.412	0.827	0.029^*^	-2.240

Values that are both bolded and underlined represent statistically significant differences ^*^p < 0.05, ^**^p < 0.01. Independent Samples t-test.

To visualize group-level differences, oxyhemoglobin (HbO) concentration curves were plotted for MDD patients and healthy controls under the three emotional auditory conditions. **Figures 4**–**6** show the results for the happy, calm, and fearful conditions, respectively. In each plot, red and blue curves represent HbO and deoxyhemoglobin (HbR) changes, and channels with statistically significant group differences are highlighted with red dashed rectangles.

**Figure 4:**
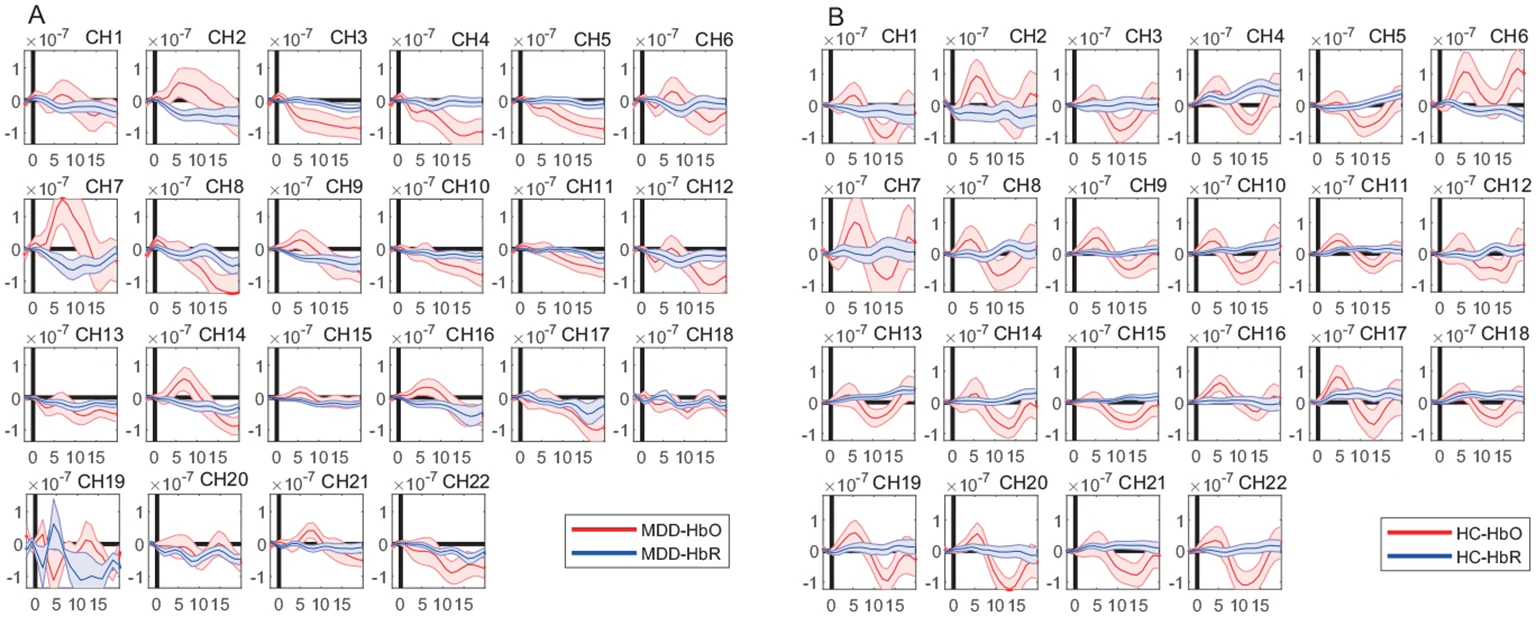
Changes in HBO concentration under the positive condition in the MDD group **(A)** and HC group **(B)**.

**Figure 5:**
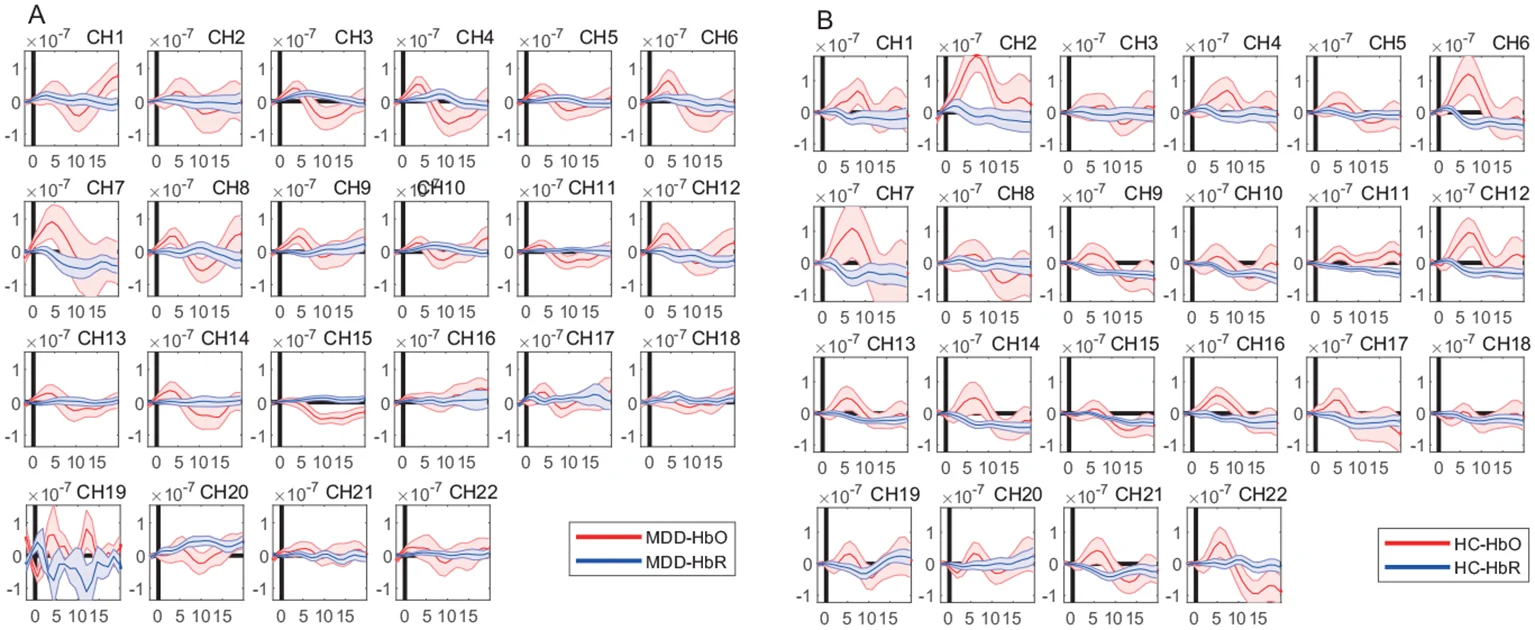
Changes in HBO concentration under the neutral condition in the MDD group **(A)** and HC group **(B)**.

**Figure 6:**
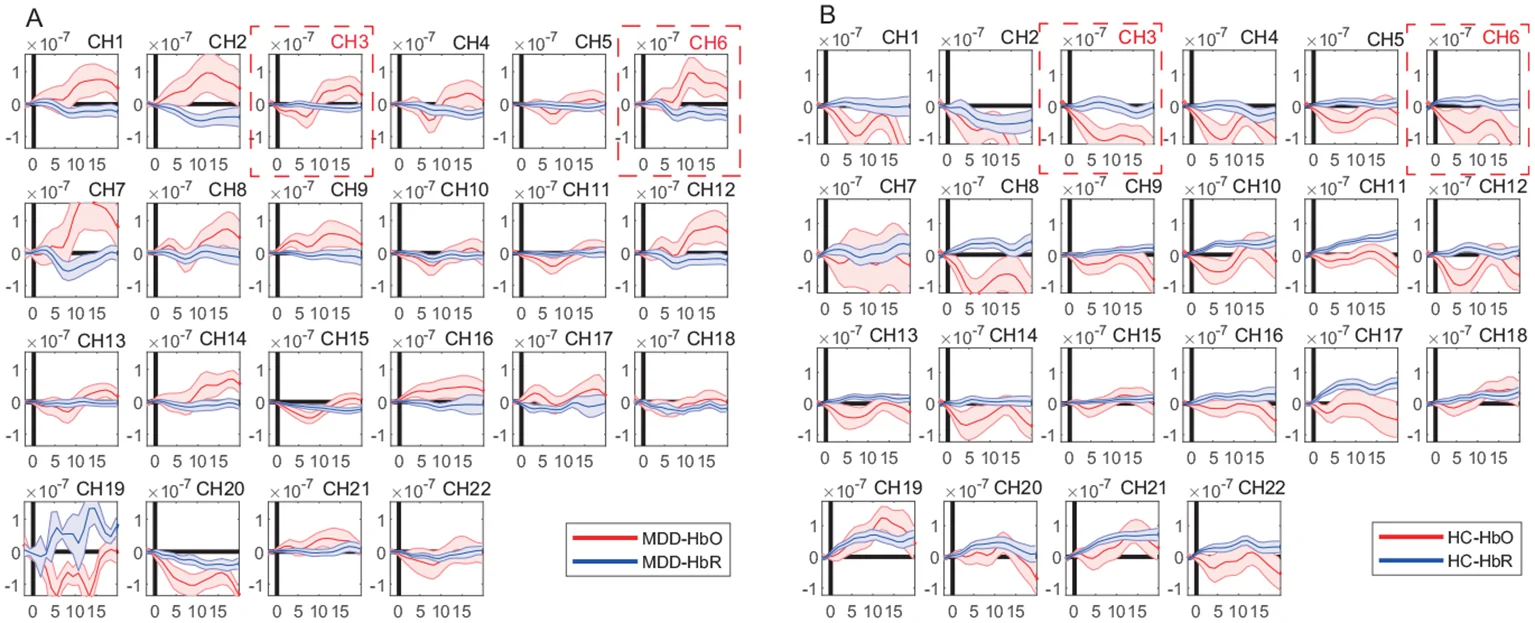
Changes in HBO concentration under the negative condition in the MDD group **(A)** and HC group **(B)**.

No significant differences were observed under the happy and calm conditions. However, under the fearful condition, CH3 and CH6 showed significant group differences. These findings suggest that fear-related auditory stimuli elicited the strongest group-level hemodynamic divergence, indicating that HbO changes in response to fear may serve as a potential biomarker for emotional dysregulation in MDD. Accordingly, fear-based stimulation may offer a promising direction for emotion-informed classification of depressive disorders.

### 3.2 Differences across emotional conditions

In contrast to the between-group analysis, within-group analysis of oxyhemoglobin (HbO) concentration was conducted in two steps. First, we examined hemodynamic responses to three emotional contrasts—positive vs. neutral, negative vs. neutral, and positive vs. negative—separately for the MDD and healthy control groups. Second, HbO concentration curves were plotted for each contrast, with significant channels annotated accordingly. This analysis aimed to assess intra-group variability in emotional processing and to identify distinct patterns of emotional reactivity in MDD patients relative to healthy controls.

(a) MDD Group: Within-group comparisons of mean oxyhemoglobin (HbO) concentrations (5–20 s window) across emotional conditions in the MDD group are summarized in **Table 4**. No significant differences were found between the happy vs. neutral or fearful vs. neutral conditions across any of the 22 channels. However, the happy vs. fearful comparison revealed significant differences in seven channels: (p=0.004^*^ t=-3.176), CH5 (p=0.029^*^ t=-2.294), CH6 (p=0.033^*^ t=-2.240), CH9 (p=0.017^*^ t=-2.523), CH12 (p=0.012^*^ t=-2.695), CH13 (p=0.035^*^ t=-2.214), and CH17 (p=0.050^*^ t=-2.049). All significant t-values were negative, indicating lower HbO concentrations under the happy condition compared to the fearful condition. This suggests that negative emotional stimuli elicited stronger prefrontal hemodynamic responses in MDD patients. The corresponding HbO curves are shown in the left panels of **Figures 7**–**9**.

**Table 4:** Differences in mean hemodynamic concentration (5–20 s) across emotional conditions within the MDD group.

Channel	Pos vs. Neul		Neg vs. Neu		Pos vs. Neg	
	*p*	*t*	*p*	*t*	*p*	*t*
CH3	0.259	-1.152	0.297	-1.152	0.004^**^	-3.176
CH5	0.224	-1.242	0.835	-1.242	0.029^*^	-2.294
CH6	0.925	-0.095	0.176	-0.095	0.033^*^	-2.240
CH9	0.537	-0.625	0.494	-0.625	0.017^*^	-2.523
CH12	0.303	-1.048	0.347	-1.048	0.012^*^	-2.695
CH13	0.449	-0.768	0.555	-0.768	0.035^*^	-2.214
CH17	0.210	-1.283	0.802	-1.283	0.050^*^	-2.049

Values that are both bolded and underlined represent statistically significant differences ^*^p < 0.05, ^**^p < 0.01, Paired Samples t-test.

**Figure 7:**
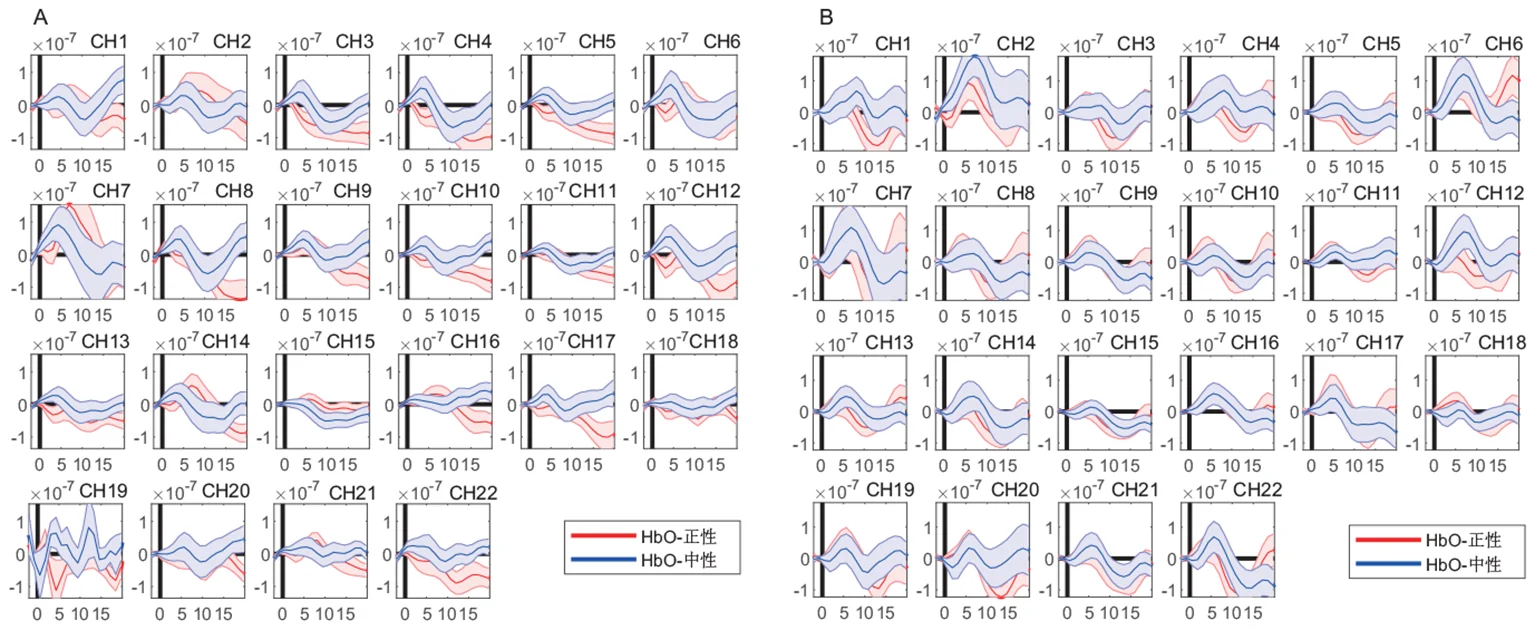
HbO concentration changes for pos vs. neu in MDD **(A)** and HC **(B)** groups.

**Figure 8:**
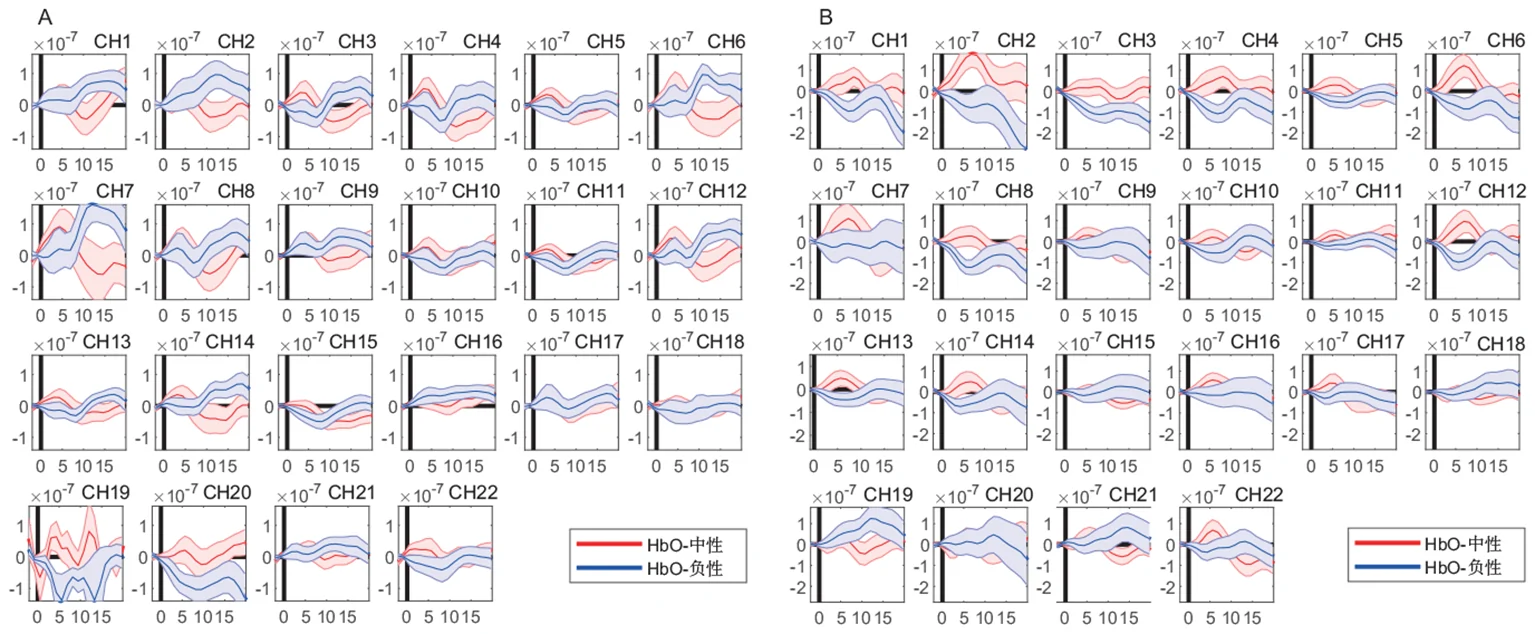
HbO concentration changes for neg vs. neu in MDD **(A)** and HC **(B)** groups.

**Figure 9:**
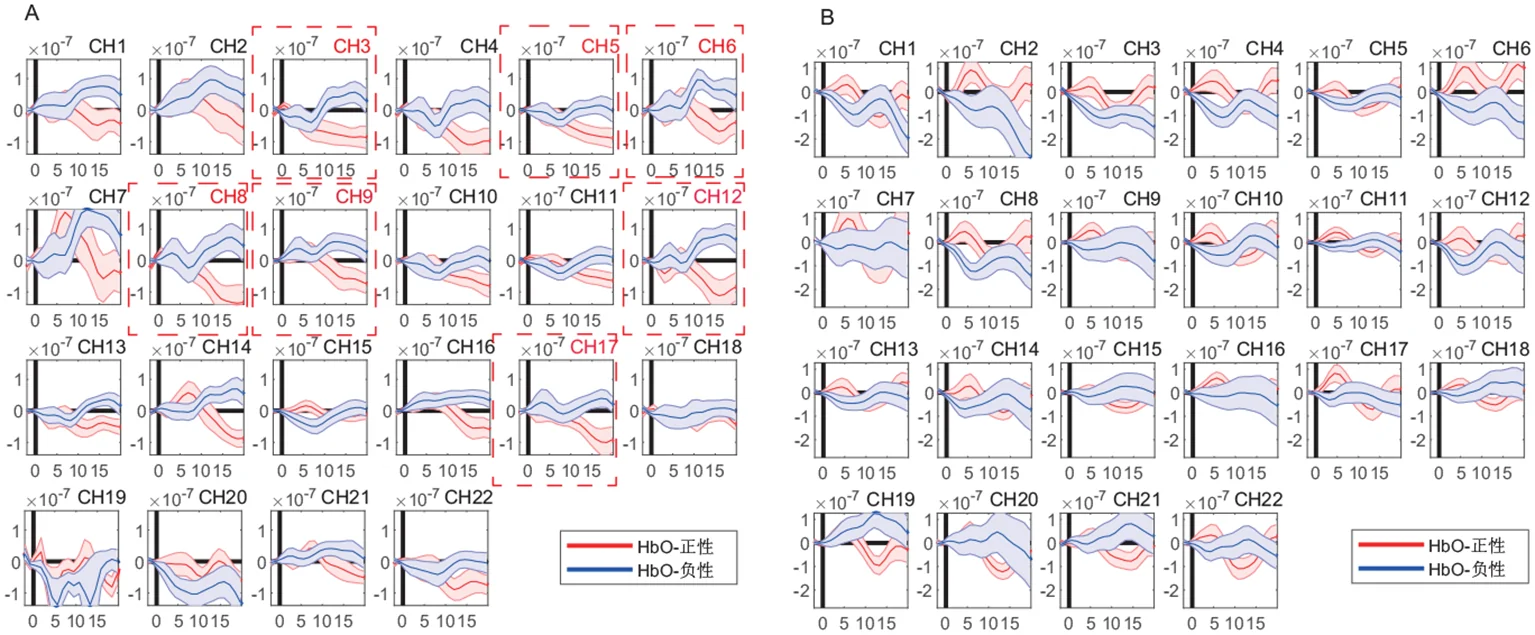
HbO concentration changes for pos vs. neg in MDD **(A)** and HC **(B)** groups.

(b) HC Group: Within-group comparisons of mean oxyhemoglobin (HbO) concentrations (5–20 s window) across emotional conditions in the HC group revealed no significant differences across any of the 22 channels for the happy vs. neutral and fearful vs. neutral contrasts. Unlike the MDD group, no significant differences were observed in the happy vs. fearful comparison either.Given that both happy and fearful stimuli are high in arousal, this result suggests that these emotions elicited comparablehemodynamic responses in healthy individuals. Additionally, polarity analysis of t-values showed a distinct trend: in the MDD group, 19 out of 22 channels exhibited negative t-values, while only 3 were positive, indicating a consistent directional pattern. In contrast, the HC group showed a more balanced distribution, with 8 negative and 14 positive *t*−values. These findings may provide further insight into altered emotional reactivity patterns in MDD.

## 4 GLM-based analysis of prefrontal activation

While mean oxyhemoglobin (HbO) concentrations offer a general overview of group differences between individuals with MDD and healthy controls, such analyses are limited to coarse channel-level interpretations and may overlook spatially specific activationpatterns. To address this limitation, a general linear model (GLM)-based activation analysis was performed to identify region-specific responses in the prefrontal cortex across emotional conditions, mapping channel-level data to anatomically defined brain regions.The corresponding procedure is shown in **Figure 10** This analysis consisted of two components: (1) Between-group comparison: Regional activation differences between MDD patients and healthy controls were examined under the same emotional condition. (2) Within-group comparison: Activation differences between emotional conditions were assessed within each group to determine emotion-specific activation profiles.

**Figure 10:**
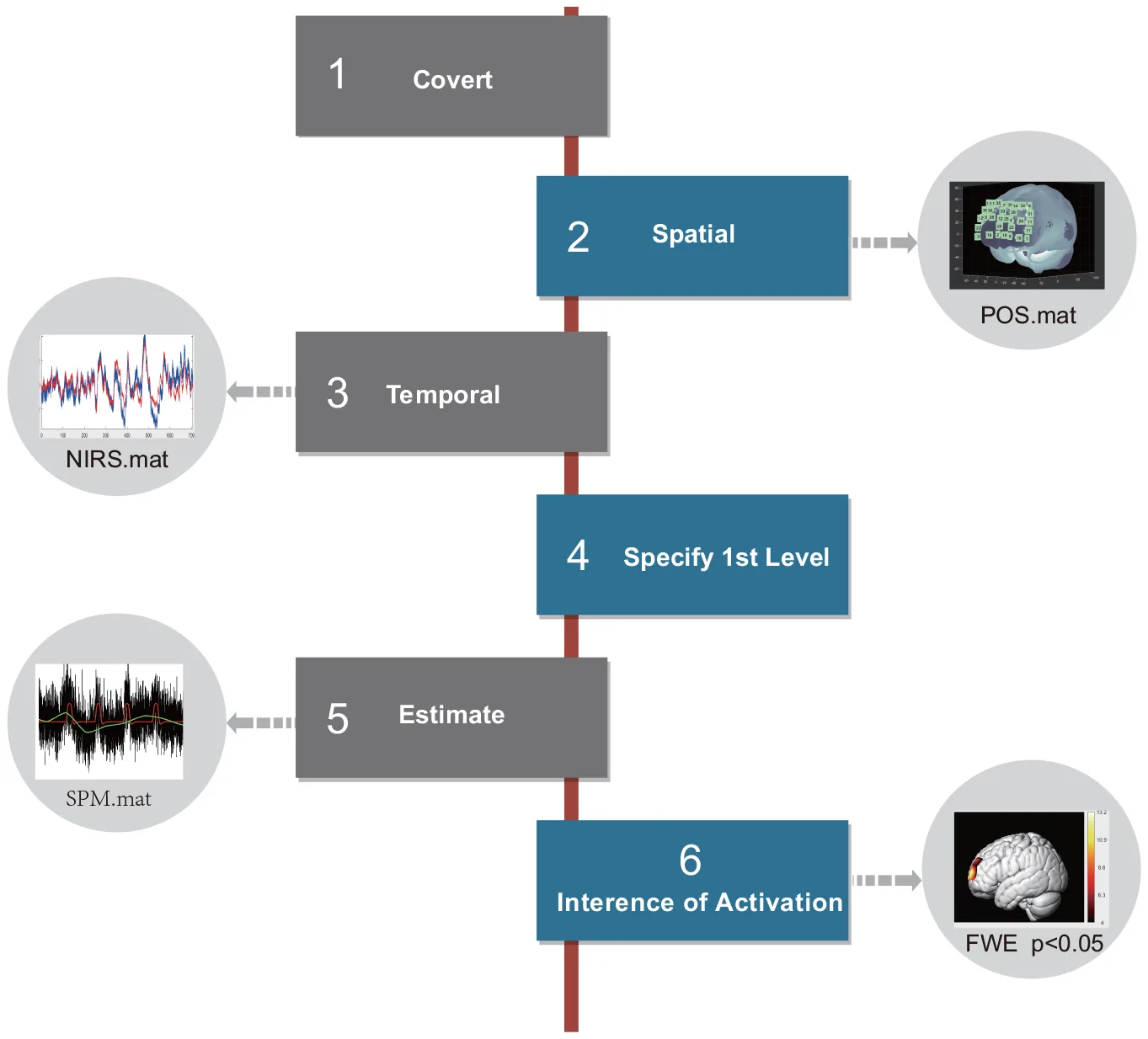
The flowchart of GLM analysis.

### 4.1 Group differences between MDD and healthy controls (HC)

In this study, the *β* values estimated by the GLM were used as activation features for subsequent channel selection and classification. The *β* value represents the regression coefficient that quantifies the magnitude of the task-evoked hemodynamic response after fitting the observed HbO signal to the experimental design matrix. Compared with directly using raw time-series signals, *β* values provide a compact summary of cortical activation while reducing the influence of temporal noise, baseline drift, and physiological fluctuations. Therefore, *β* values have been widely adopted in task-based fNIRS studies as robust biomarkers for statistical analysis and machine learning classification.

The between-group comparison of GLM-derived *β* values across emotional conditions is summarized in **Table 5**. No significant activation differences were observed between the MDD and healthy control groups under the happy condition across any of the 22 channels. Under the neutral condition, significant group differences emerged in two channels: CH2 (p=0.013^*^ t=-2.574) and CH4 (p=0.039^*^ t=-2.112). All *t* values for this condition were negative, indicating consistently lower activation in the MDD group compared to controls. The mechanism underlying this suppression remains unclear and warrants further investigation. In contrast, under the fearful condition, four channels showed significant between-group differences: CH1 (p=0.039^*^ t=2.19), CH3 (p=0.026^*^ t=-2.281), CH9 (p=0.037^*^ t=2.139), and CH16 (p=0.020^*^ t=2.389). Notably, three of these four channels showed positive *t* values, suggesting greater prefrontal activation in MDD patients relative to controls in response to fear-related stimuli.

**Table 5:** Differences in mean hemodynamic concentration (5–20 s) across emotional conditions within the MDD group.

Channel	Pos		Neu		Neg	
	p	t	p	t	p	t
CH1	0.858	0.180	0.657	-0.446	0.039^*^	2.109
CH2	0.571	0.570	0.013^*^	-2.574	0.168	1.396
CH3	0.516	0.654	0.802	-0.252	0.026^*^	2.281
CH4	0.676	0.420	0.039^*^	-2.112	0.174	1.376
CH9	0.639	0.472	0.304	-1.036	0.037^*^	2.139
CH16	0.583	0.552	0.081	-1.775	0.020^*^	2.389

Values that are both bolded and underlined represent statistically significant differences ^*^p < 0.05, ^**^p < 0.01, Paired Samples t-test.

To facilitate intuitive interpretation of the results, activation values from significant channels were visualized using overlaid boxplots and scatterplots, as shown in **Figures 11** and **12**. **Figure 11** illustrates the distribution of activation values under the neutral condition for both the MDD and HC groups. The median activation in the MDD group was lower than in the HC group, indicating reduced average activation. Moreover, the narrower interquartile range in the MDD group suggests a more concentrated distribution of activation values relative to the broader variability observed in the HC group. **Figure 12** illustrates the distribution of prefrontal activation values under the negative condition for both groups. In contrast to the neutral condition, the MDD group exhibited a higher median activation value than the HC group, indicating that prefrontal activation in response to negative stimuli was markedly greater in individuals with MDD. The interquartile range pattern was consistent with that observed under the neutral condition, suggesting that the distribution of activation values in the MDD group remained more concentrated. This result indicates that while emotional condition modulates the level of activation, it does not substantially alter the overall distribution characteristics of the activation response.

**Figure 11:**
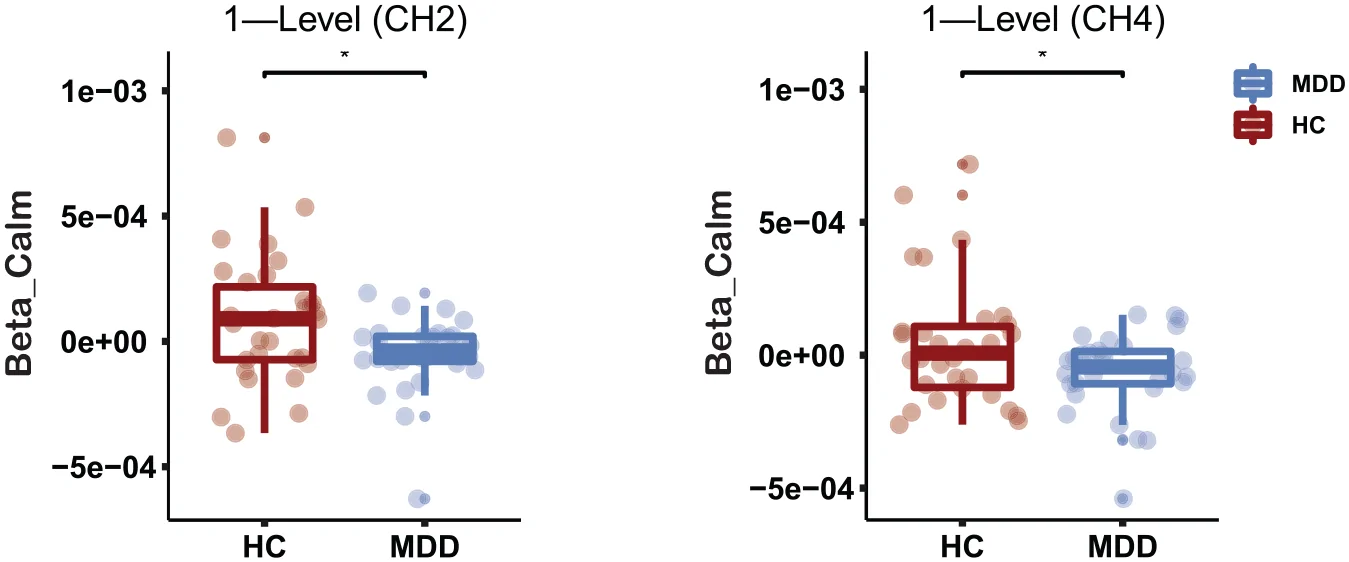
Significant channels with between-group differences (MDD vs. HC) under the neutral condition.

**Figure 12:**
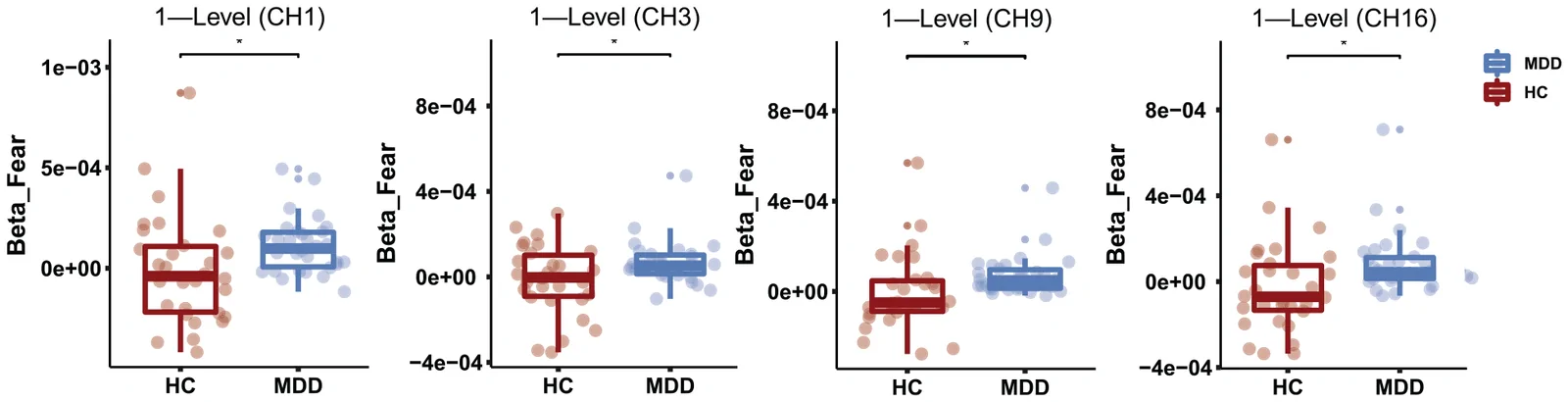
Significant channels with between-group differences (MDD vs. HC) under the negative condition.

The analyses described above focused primarily on activation differences at the channel level. To further localize these effects to anatomically defined cortical regions, group-level activation differences under the three emotional conditions were mapped from scalp channels to cortical space.Specifically, scalp-level MNI coordinates were projected onto the cortical surface using the mni_scalp2cortex function (33). Next, the nirs2img function (nirs2img(imgFileName, mni_new, value, 1, 0, 0)) was used to combine *t* values and MNI coordinates into NIfTI (.nii) files compatible with standard neuroimaging tools. The resulting cortical activation maps were visualized using the BrainNet Viewer toolbox (34). The specific cortical mapping results are presented below.

**Figure 13** illustrates the topographic distribution of prefrontal cortex activation in MDD patients and healthy controls under the happy, neutral, and fearful conditions. Under positive stimulation, significant activation differences were observed in the left dorsolateral superior frontal gyrus (SFGdor.L), with healthy controls exhibiting greater activation than MDD patients.In the neutral condition, activation differences were more diffusely distributed, and no specific region reached statistical significance. In contrast, under negative stimulation, significant group differences re-emerged in the same region (SFGdor.L), suggesting its consistent involvement in processing emotionally salient stimuli. Notably, the direction of activation was reversed: MDD patients showed greater activation than controls under the fearful condition.

**Figure 13:**
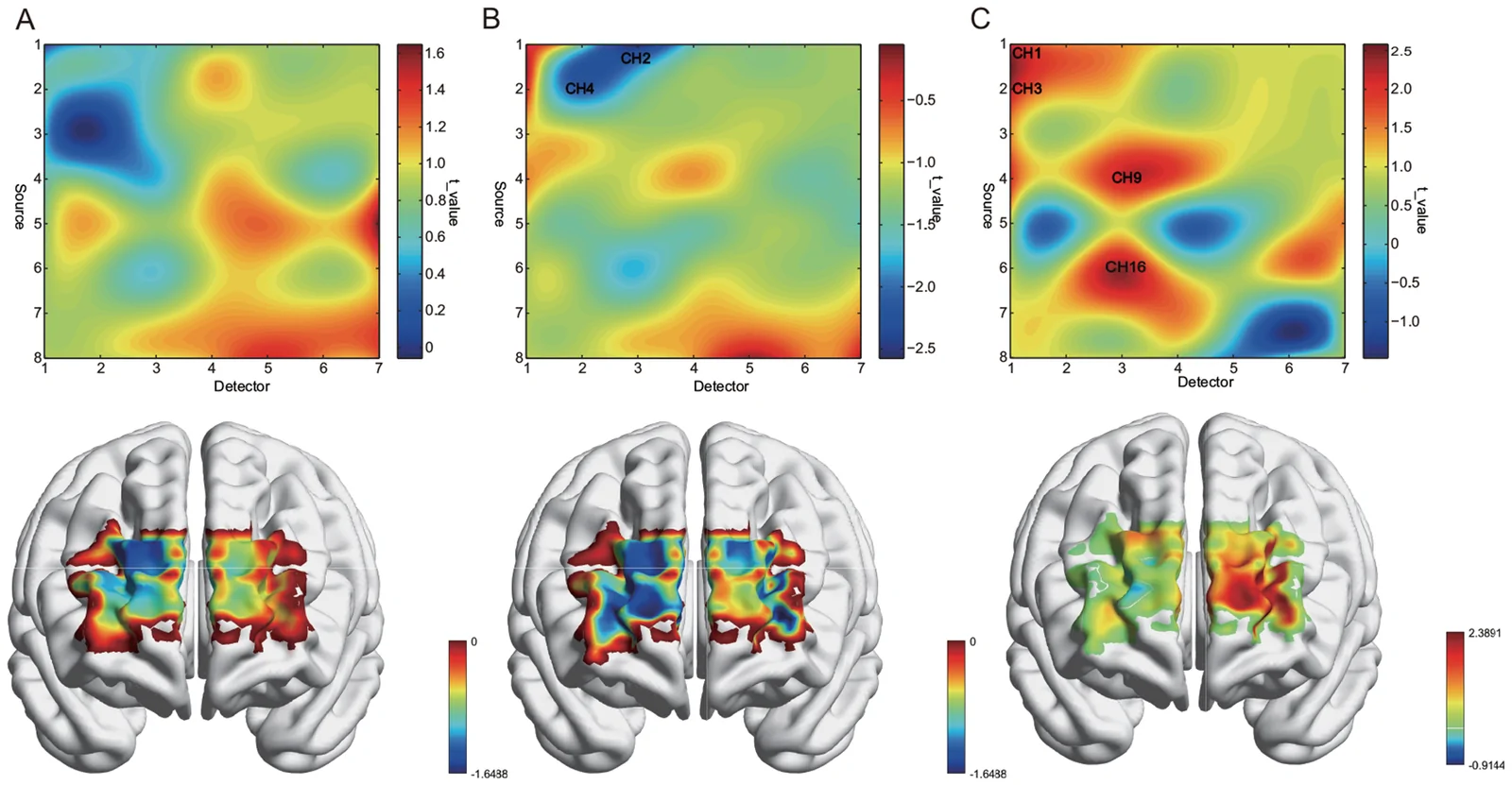
Prefrontal activation differences between MDD and HC groups under the three condition: **(A)** Happy, **(B)** Neutral, **(C)** Fearful.

### 4.2 Emotion-related differences

Consistent with the mean oxyhemoglobin (HbO) concentration analysis, within-group activation differences across the three emotional conditions were also examined separately for the MDD and healthy control groups.

(a) MDD Group: Within-group analysis of GLM-derived *β* values for MDD patients under different emotional conditions is summarized in **Table 6**. No significant activation differences were found between the happy and neutral conditions. However, comparison between the fearful and neutral conditions revealed significant differences in 17 of the 22 channels, spanning most regions of the prefrontal cortex. This result suggests that negative emotional stimuli elicited widespread and robust prefrontal activation in individuals with MDD. Additionally, the happy vs. fearful comparison showed significant activation differences in 5 channels.

**Table 6:** Differences in activation *β* values across emotional conditions in the MDD group.

Channel	Pos vs. Neul		Neg vs. Neu		Pos vs. Neg	
	p	t	p	t	p	t
CH1	0.425	0.809	0.003^**^	-3.295	0.030^*^	-2.276
CH2	0.068	1.894	0.005^*^	-3.066	0.047^*^	-2.077
CH3	0.172	1.400	0.001^***^	-3.870	0.007^**^	-2.889
CH4	0.427	0.806	0.009^**^	-2.798	0.036^*^	-2.199
CH5	0.248	1.179	0.021^*^	-2.433	0.124	-1.583
CH6	0.893	0.135	0.036^*^	-2.203	0.079	-1.820
CH7	0.183	1.365	0.004^**^	-3.134	0.101	-1.693
CH8	0.288	1.083	0.004^**^	-3.081	0.013^*^	-2.631
CH9	0.517	0.656	0.019^*^	-2.478	0.012^**^	-2.671
CH10	0.404	0.847	0.019^*^	-2.475	0.056	-1.994
CH12	0.555	0.597	0.015^*^	-2.586	0.121	-1.596
CH13	0.177	1.384	0.029^*^	-2.295	0.249	-1.176
CH14	0.099	1.704	0.002^**^	-3.447	0.054	-2.010
CH15	0.322	1.008	0.004^**^	-3.140	0.032^*^	-2.260
CH16	0.213	1.272	0.009^**^	-2.795	0.049^**^	-2.059
CH17	0.237	1.207	0.013^*^	-2.650	0.121	-1.595
CH18	0.237	1.207	0.035^*^	-2.217	0.125	-1.580

Values that are both bolded and underlined represent statistically significant differences ^*^p < 0.05, ^**^p < 0.01, paired Samples t-test.

Given the large number of significant channels identified in the contrast between negative and neutral conditions, the results are visualized in **Figure 14** using overlaid boxplots and scatterplots. The plots show changes in median and interquartile range of activation values in MDD patients under both conditions. Compared to neutral stimuli, negative stimuli elicited higher median activation, indicating stronger overall prefrontal activation. Additionally, the interquartile range was narrower under the negative condition, suggesting a more concentrated distribution of responses.

**Figure 14:**
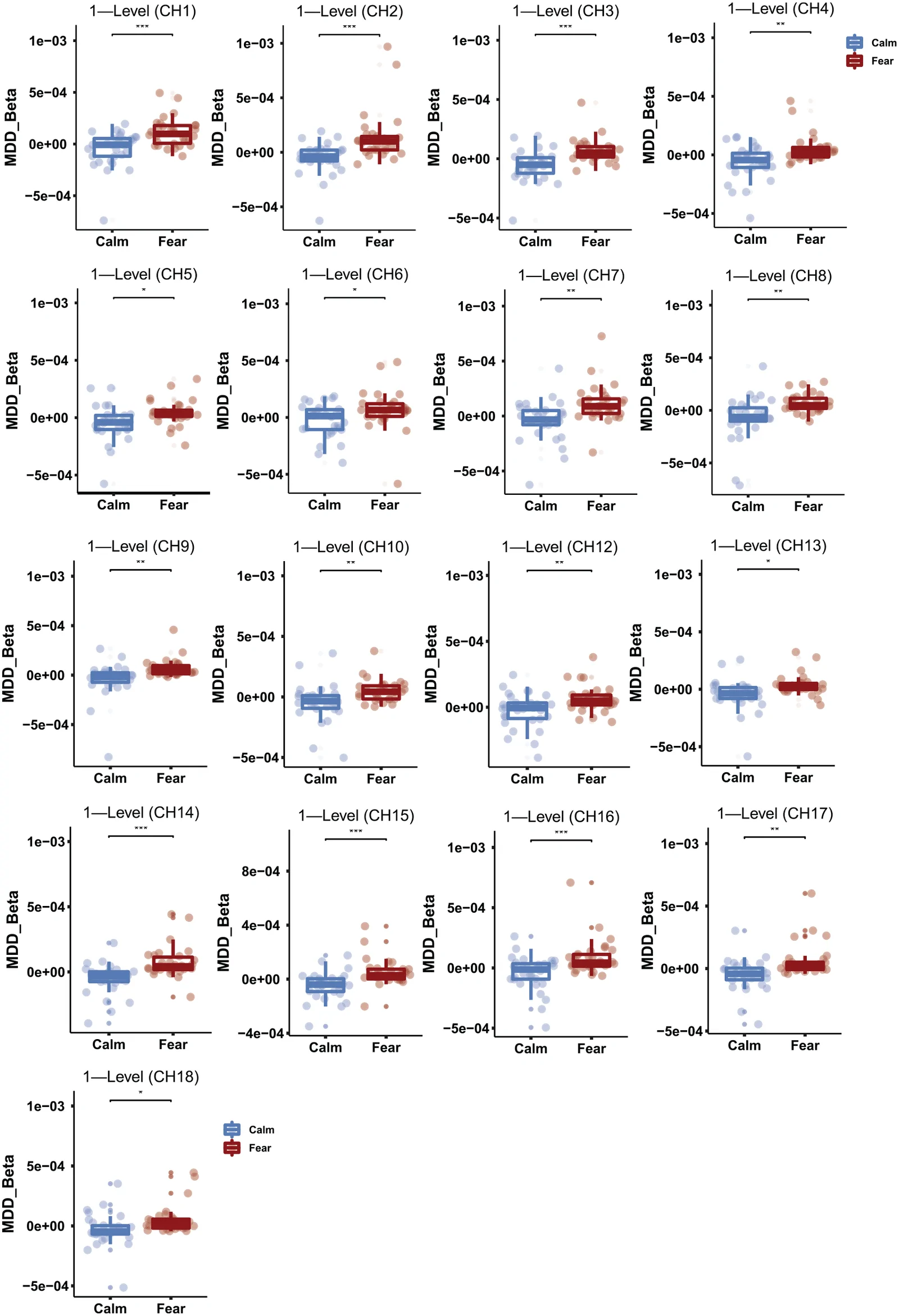
Prefrontal activation differences between MDD and HC groups under the neg condition. *p < 0.05, **p < 0.01, ***p < 0.001.

**Figure 15** displays prefrontal cortical activation topographies for the MDD group across the three within-group emotional contrasts. No distinct activation differences were observed between the positive and neutral conditions. In the neutral vs. negative contrast, increased activation was observed predominantly in the right prefrontal cortex, particularly in the right middle frontal gyrus (MFG.R) and the dorsolateral superior frontal gyrus bilaterally (SFGdor.L and SFGdor.R). However, the highest*t* values were found in the left dorsolateral superior frontal gyrus (SFGdor.L), indicating this region exhibited the strongest activation. Similarly, the positive vs. negative comparison also revealed significant activation differences localized to SFGdor.L. This finding is consistent with the between-group analysis, in which the same region showed significant group differences under negative emotional stimulation, further emphasizing the critical role of the left dorsolateral prefrontal cortex in MDD patients’ neural responses to negative affect.

**Figure 15:**
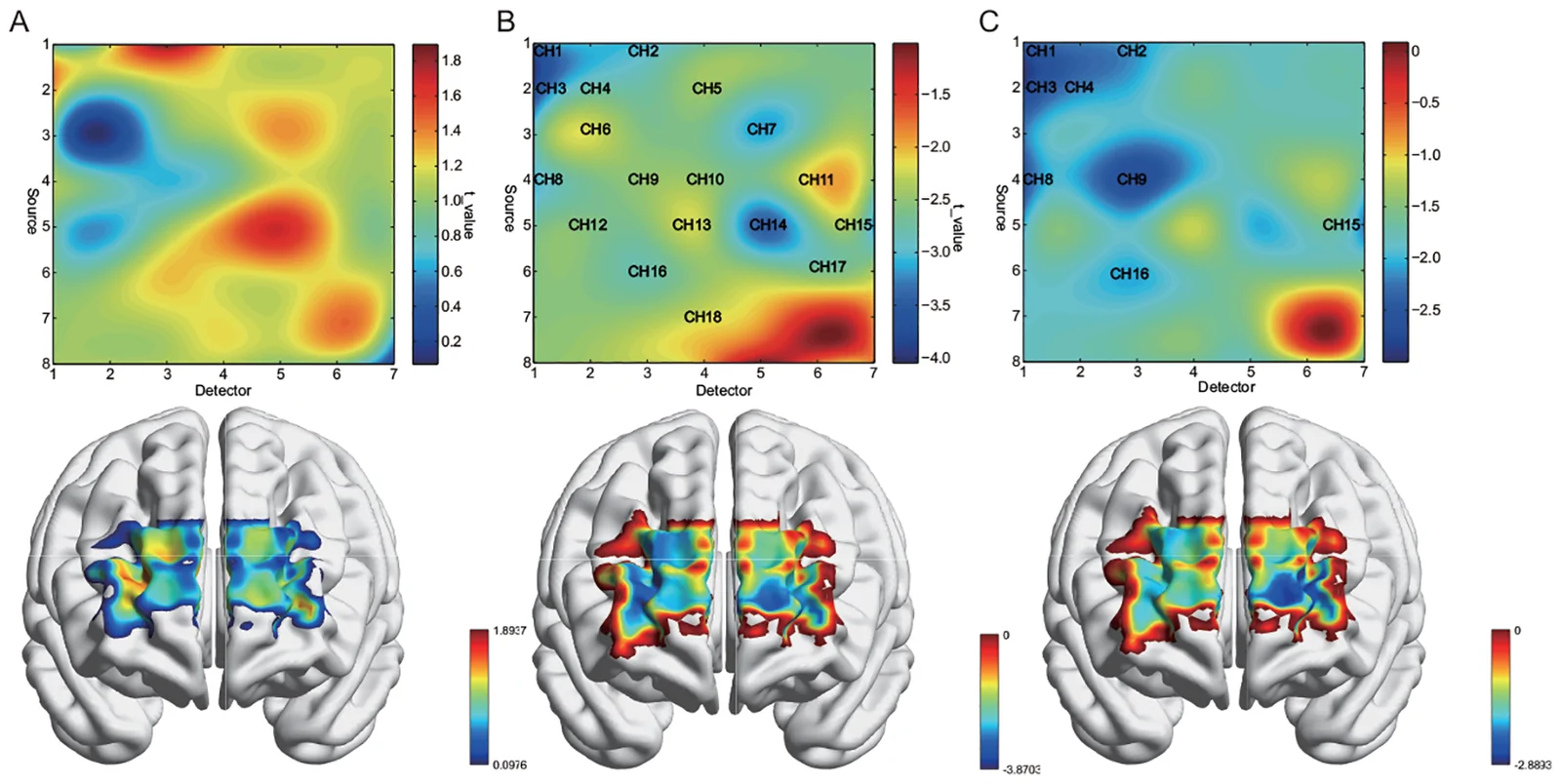
Activation difference channels for MDD under different emotional contrast: **(A)** Happy vs. Neutral, **(B)** Fearful vs. Neutral, **(C)** Happy vs. Fearful.

**Table 7:** Simple neural network structure and parameters.

BP	Input layer	No. of hidden layers	No. of hidden layer neurons	Output layer
MNN	22	5	[10, 10, 10, 10, 10]	2
FFNN	22	3	[10, 10, 10]	2
CFNN	22	2	[10, 10]	2
RNN	22	4	[10, 10, 10, 10]	2

(b) HC Group: Within-group comparisons of GLM-derived *β* values across emotional conditions for the healthy control (HC) group are summarized in **Figure 16**. In contrast to the MDD group, no significant activation differences were observed in any of the three emotional contrasts. The prefrontal activation topographies reveal minimal differences across conditions, with no consistently distinct patterns emerging in specific brain regions.These results suggest that, in healthy individuals, activation-based measures alone may be insufficient to capture emotion-related neural changes. More advanced approaches—such as brain network analysis or dynamic causal modeling—may be needed to reveal the underlying neural dynamics associated with emotional processing in non-clinical populations.

**Figure 16:**
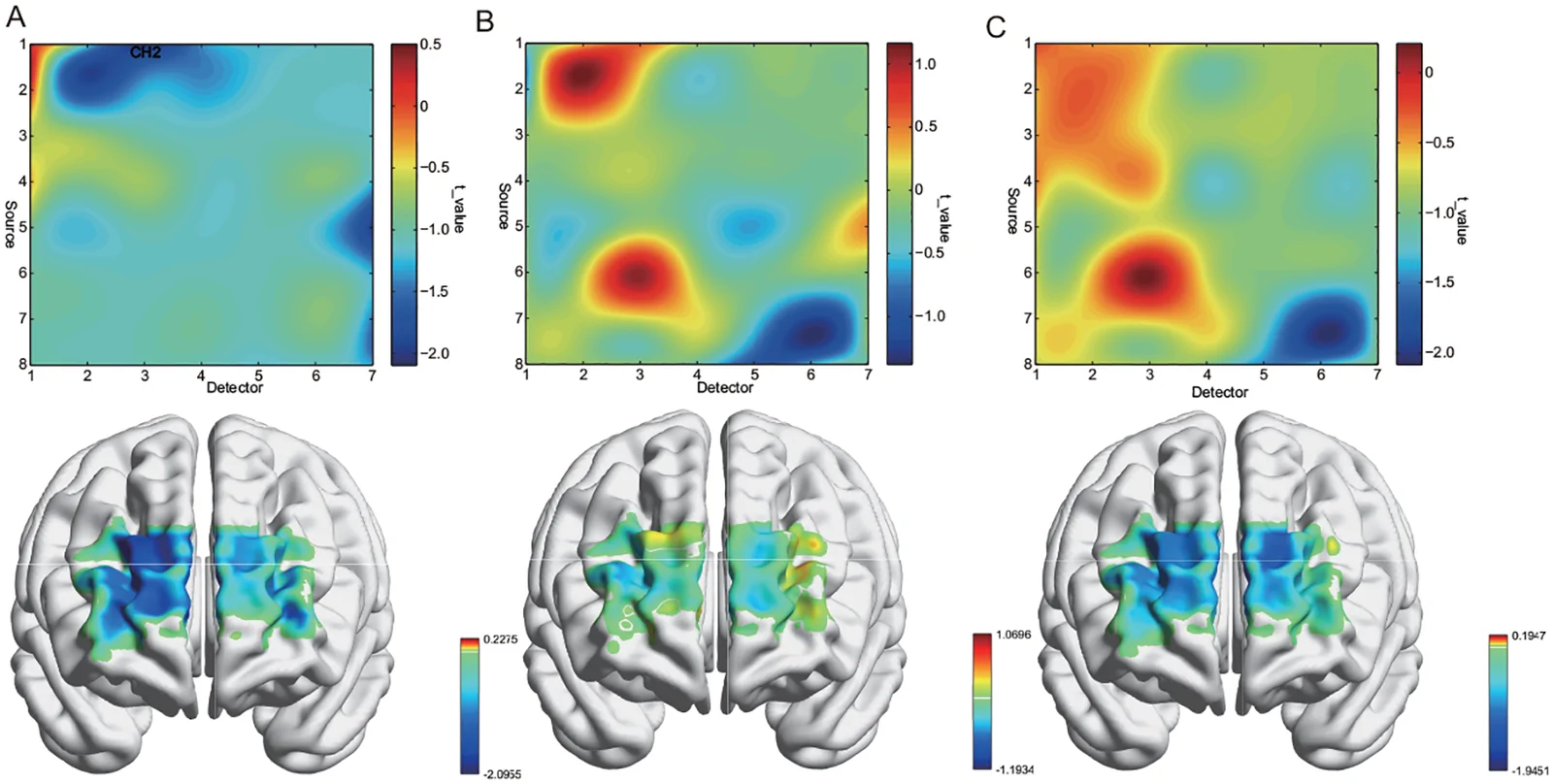
Activation difference channels for HC under different emotional contrast: **(A)** Happy vs. Neutral, **(B)**Fearful vs. Neutral, **(C)** Happy vs. Fearful.

## 5 *β*_value based analysis of emotion recognition in MDD patients

The Back Propagation (BP) neural network is a widely used multilayer feedforward architecture trained via the error backpropagation algorithm. As one of the most extensively applied neural network models, the BP network has gained considerable attention in various domains, including brain disorder classification. In this study, the dataset was split into training, validation, and testing sets in a 70:15:15 ratio. The Levenberg–Marquardt (L-M) algorithm was employed as the training function, and model performance was assessed using the mean squared error (MSE), where lower values indicate higher prediction accuracy. The specific parameters are shown in the **Table 7**.

### 5.1 Results of classification

The data is divided into three parts: a training set, a validation set, and a test set, with the specific division ratios as follows: trainRatio = 0.7, valRatio = 0.15, testRatio = 0.15. The Levenberg-Marquardt (L-M) algorithm is used as the training function, and the Mean Squared Error (MSE) is employed as the quantitative metric to evaluate the prediction performance of the BP neural network. A smaller MSE value indicates higher model accuracy.

**Table 8** presents the classification results for MDD and HC groups under the three emotional conditions using *β*_value features. The highest classification accuracies achieved were 91.60% for the happy condition, 90.00% for the neutral condition, and 93.33% for the fearful condition. Notably, the fearful condition also yielded the lowest standard deviation (3.43), indicating higher classification stability. Among the four neural network models evaluated, performance under the fearful condition consistently outperformed the other two conditions. These findings suggest that negative emotional stimuli elicit more discriminative neural activation patterns between MDD patients and healthy controls. Therefore, *β*_values derived from the fearful condition may serve as potential neural biomarkers for the classification of MDD.

**Table 8:** Classification accuracy for MDD and HC groups across different emotional conditions.

BP	Happy (%)	Calm (%)	Fear (%)
MNN	71.67	78.33	85.00
FFNN	80.00	80.00	90.00
CFNN	81.66	73.33	93.33
RNN	91.66	90.00	93.33
***M*** ± ***SD***	81.25 ± 8.20	80.41 ± 6.99	89.58 ± 3.43

Bold and underlined values represent the optimal results for features under different emotional conditions.

**Table 9** summarizes the classification results for distinguishing between happy and fearful emotional conditions within the MDD and HC groups. The highest classification accuracy for the MDD group was 85.00%, while the HC group achieved a peak accuracy of 88.33%. However, when averaging across all models, the MDD group exhibited a higher mean accuracy (80.33%) compared to the HC group (77.91%). Moreover, the MDD group showed a lower standard deviation (8.20), indicating more consistent classification performance. These findings suggest that individuals with MDD demonstrate more distinct neural differences between positive and negative emotional states, making emotional condition classification more robust within this group than in healthy controls.

**Table 9:** Classification accuracy of MDD and HC groups for positive and negative emotions.

BP	MDD group (%)	HC group(%)
MNN	73.33	66.67
FFNN	83.33	**88.33**
CFNN	80.00	85.00
RNN	**85.00**	71.67
*M* ± *SD*	81.16 ± 5.89	77.90 ± 10.40

Bold and underlined values represent the optimal results for features under different emotional conditions.

In psychiatric diagnostic studies, researchers often face the challenge of small sample sizes coupled with high-dimensional data, which can introduce learning bias in classification tasks. To address this issue and identify the optimal channel subset for binary classification, this study employed the ReliefF feature selection algorithm to evaluate *β*_value features. The objective was to achieve a minimal feature set with maximal classification performance.ReliefF is a feature weighting algorithm originally proposed by Kira (35–37). It assigns weights to individual features based on their relevance to class labels, retaining those with high discriminative power and discarding those below a predefined threshold. By selecting a subset of highly relevant features, ReliefF reduces dimensionality and improves classifier performance. The algorithm is computationally efficient, scalable, and applicable to various data types, making it particularly suitable for high-dimensional neuroimaging data.

**Figure 17** presents the ranked feature weights of all channels under the three emotional conditions. Panels A, B, and C show the weight distributions for the happy, neutral, and fearful conditions, respectively. Under the happy condition, 40% of channels exhibited negative feature weights, and 18% did so under the neutral condition, suggesting that these channels may have contributed negatively to classification performance. In contrast, all channels under the fearful condition showed positive feature weights, indicating a more uniformly informative feature set for classification. Based on these findings, the relationship between the number of selected channels and classification accuracy was further investigated to identify an optimal balance between feature dimensionality and model performance.

**Figure 17:**
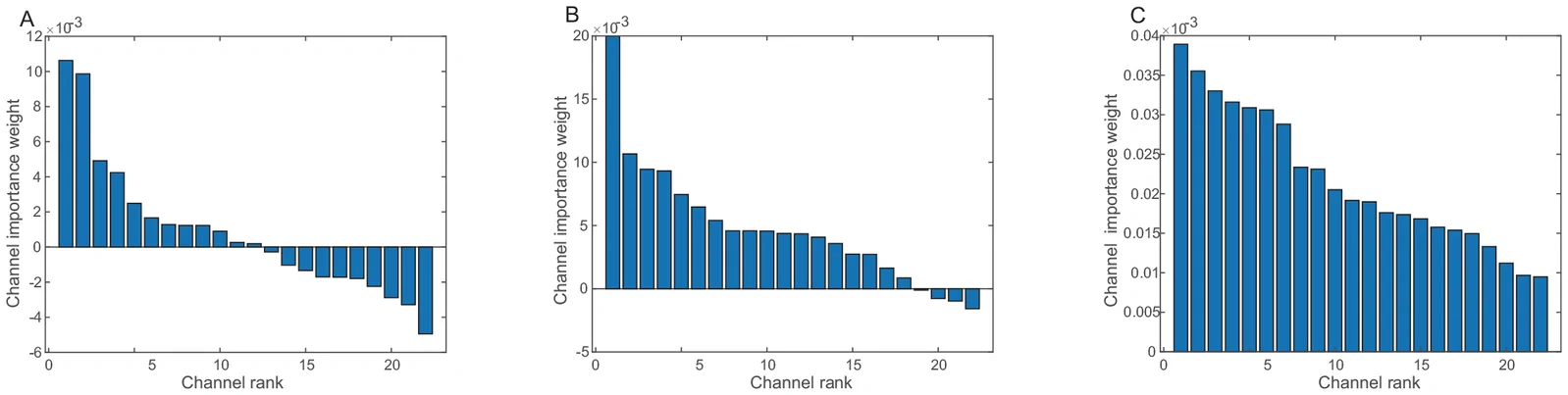
Ranked feature weights of all channels under the three emotional conditions: **(A)** Happy, **(B)** Neutral, **(C)** Fearful.

**Table 10** presents the classification results using β-value features under varying feature dimensionalities. Ref_5, Ref_10, Ref_15, and Ref_20 represent results obtained when the number of selected features was reduced to 5, 10, 15, and 20,respectively, while “All” denotes performance using all 22 features. To visually illustrate the relationship between feature dimensionality and classification performance, the results in **Table 9** are plotted as line graphs in **Figures 18**–**20**, with the optimal feature dimension indicated by a black dashed line.

**Table 10:** Classification accuracy of MDD and HC groups based on β features under different emotional stimuli.

FD	Happy (%)					Calm (%)					Fear (%)				
	MNN	FFNN	CFNN	RNN	*M* ± *SD*	MNN	FFNN	CFNN	RNN	*M* ± *SD*	MNN	FFNN	CFNN	RNN	*M* ± *SD*
Ref_5	70.00	83.33	85.00	75.00	78.33 ± 7.0	75.00	71.67	68.33	68.33	70.83 ± 2.7	76.67	93.33	90.00	83.33	85.83 ± 6.3
Ref_10	71.66	88.33	81.67	56.67	74.58 ± 11.92	71.67	78.33	81.67	85.00	79.17 ± 4.93	86.67	91.67	90.00	90.00	89.58 ± 1.81
Ref_15	65.00	68.33	88.33	68.33	72.49 ± 9.24	68.33	71.67	78.33	71.67	72.50 ± 3.63	80.00	91.67	88.33	90.00	87.50 ± 4.49
Ref_20	61.67	90.00	76.67	68.33	74.16 ± 10.57	78.33	78.33	80.00	90.00	81.66 ± 4.86	83.33	91.67	96.66	95.00	91.67 ± 5.14
All	71.67	80.00	81.66	91.66	81.25 ± 7.10	78.33	80.00	73.33	90.00	80.41 ± 6.05	85.00	90.00	93.33	93.33	90.42 ± 3.41

FD represents the feature dimension, and “All” refers to all dimensions, i.e., 60×484, *M* ± *SD* represents the mean and standard deviation of each feature selection dimension across the four neural networks. Bold and underlined values indicate the optimal results for features under different emotional conditions.

**Figure 18:**
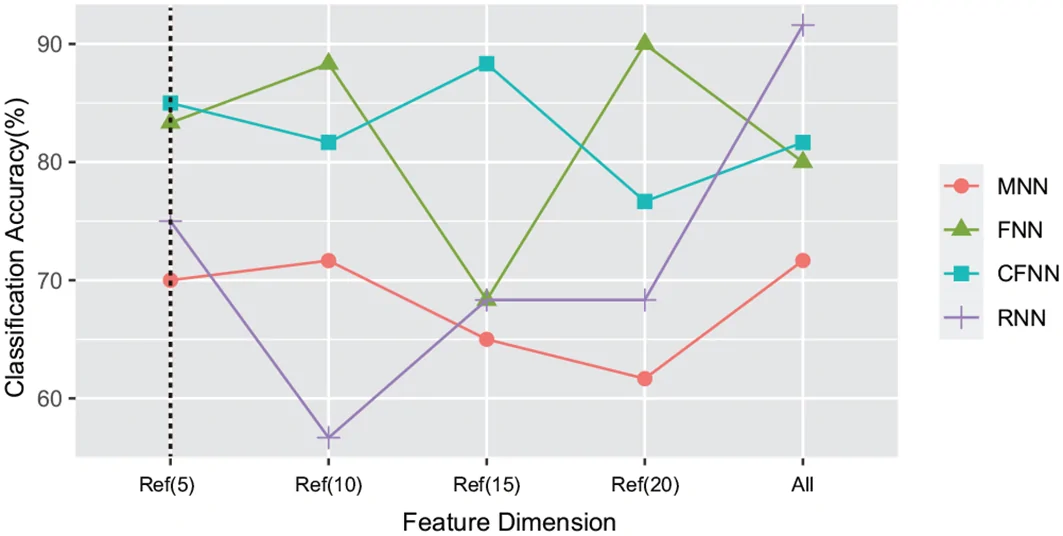
Relief F-selected optimal features for the pos condition.

**Figure 19:**
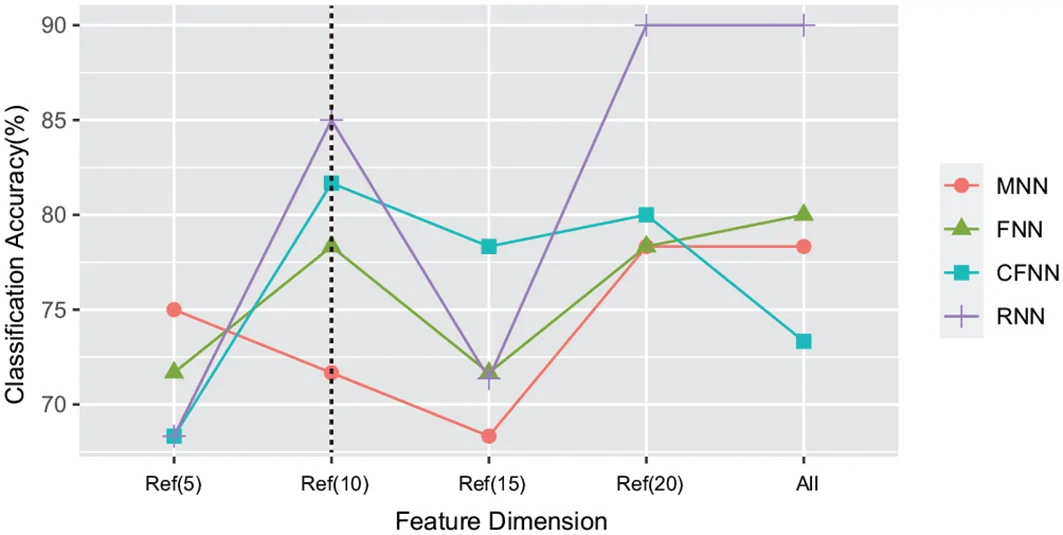
Relief F-selected optimal features for the neu condition.

**Figure 20:**
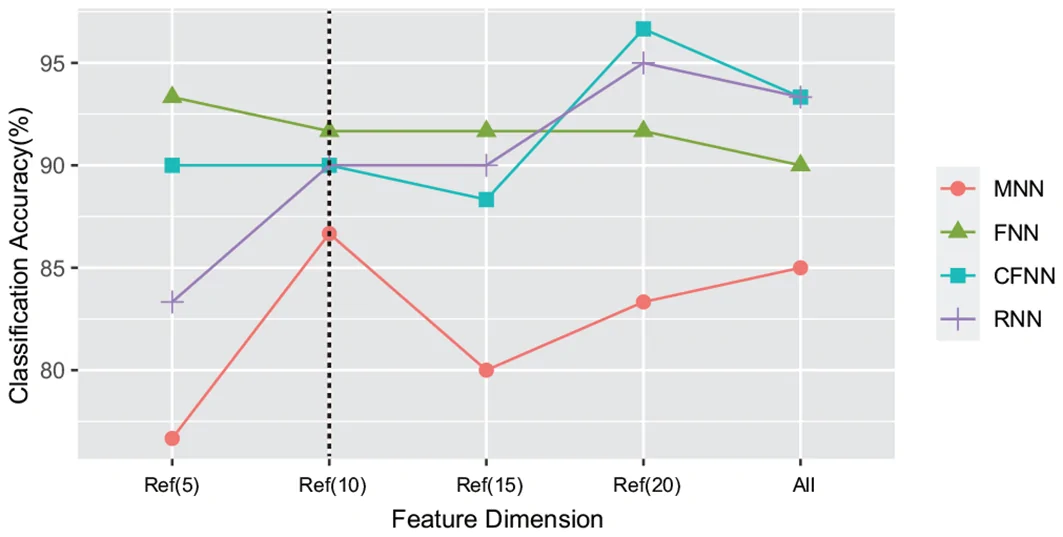
Relief F-selected optimal features for the neg condition.

The results show that under the happy condition, a classification accuracy of 78.33% ± 7.07% was achieved using only 5 features, comparable to the performance using all features (81.25% ± 7.10%).

For the neutral and fearful conditions, the highest accuracies were obtained with 10 features, reaching 79.17% ± 4.93% (All: 80.41% ± 6.05%) and 89.58% ± 1.81% (All: 90.42% ± 3.41%), respectively. Notably, classification under the fearful condition consistently yielded the highest accuracy and lowest variance across feature dimensions. These findings further support that β-value features derived from negative emotional stimulation provide stronger discriminative power for distinguishing MDD patients from healthy controls.

The ranked channel importance for each emotional condition is summarized in **Table 11**. Under the happy condition, channels 22, 15, 20, 21, and 7 were the most influential for classification. For the neutral condition, the top contributing channels included 2, 15, 7, 4, 6, 21, 12, 22, 3, and 13. In the fearful condition, channels 9, 8, 11, 16, 15, 1, 2, 10, 17, and 6 showed the greatest contribution to model performance. These results indicate that channel-level importance varies by emotional context, with specific channels showing greater discriminative relevance depending on the type of stimulus. In contrast, channels with consistently low feature weights may offer limited diagnostic value and could introduce noise or irrelevant variability intothe classification model, particularly if their activity is weakly associated with depressive pathology.

**Table 11:** Ranked channels with significant differences between MDD and HC groups under different emotional conditions.

Emotion	Channel weight ranking
Pos	**CH22**, **CH15**, **CH20**, **CH21**, **CH7**, CH19, CH14
	CH9, CH10, CH13, CH18, CH17, CH12, CH2
	CH5, CH6, CH16, CH3, CH8, CH1, CH11, CH4
Neu	**CH2**, **CH15**, **CH7**, **CH4**, **CH6**, **CH21**, **CH12**
	**CH22**, **CH3**, **CH13**, CH20, CH14, CH16, CH10
	CH8, CH5, CH17, CH1, CH11, CH9, CH19, CH18
Neg	**CH9**, **CH8**, **CH11**, **CH16**, **CH15**, **CH1**, **CH2**
	**CH10**, **CH17**, **CH6**, CH3, CH22, CH20, CH7
	CH4, CH19, CH18, CH5, CH21, CH14, CH13, CH12

Bold values indicate the optimal channel(s) for each emotional condition.

**Figure 21** presents the ranked feature weights of all channels for classifying positive versus negative emotional states in both the MDD and HC groups. A comparison between panels A and B reveals a notable difference in channel contribution patterns: in the MDD group, the majority of channels contributed meaningfully to the classification model, whereas in the HC group, only a limited number of channels—primarily five—showed substantial influence.

**Figure 21:**
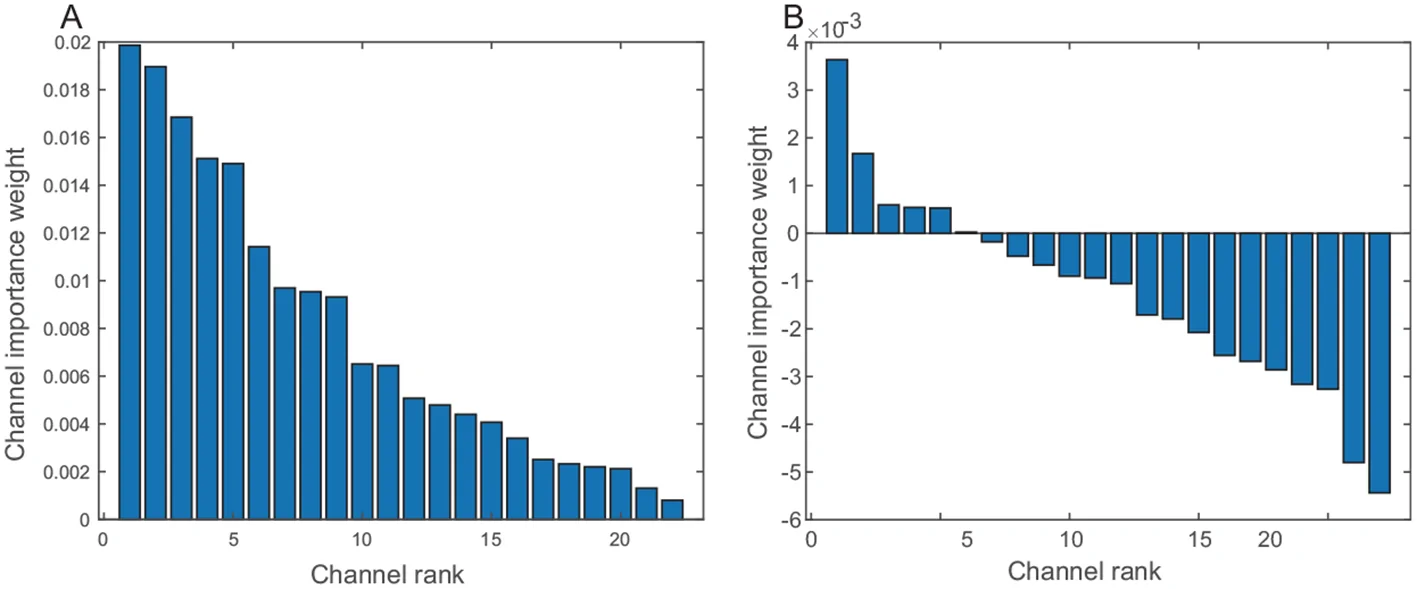
Channel Weight ranking across positive versus negative emotional conditions: **(A)** MDD group, **(B)** HC group.

**Table 12** summarizes the classification results using *β*-value features under different feature dimensionalities. In the MDD group, reducing the feature set to five dimensions maintained relatively high performance, with an average accuracyof 79.58% ± 4.38% across the four neural network models, comparable to the result using all features (79.58% ± 5.51%). In contrast, the HC group achieved only 70.00% ± 8.38% accuracy with five features, slightly improving to 70.00% with ten features, but still below the performance using all features (74.16% ± 13.08%). Additionally, the MDD group consistently showed lower standard deviations across conditions, indicating more stable classification outcomes compared to the HC group. These findings suggest that individuals with MDD exhibit greater neural discriminability between positive and negative emotional states, indicating a more pronounced differentiation in emotional valence processing compared to healthy controls.

**Table 12:** Classification accuracy of MDD and HC groups for positive vs. negative emotions.

FD	MDD (%)					HC (%)				
	MNN	FFNN	CFNN	RNN	*M* ± *SD*	MNN	FFNN	CFNN	RNN	*M* ± *SD*
Ref_5	73.33	80.00	83.33	81.67	79.58 ± 4.38	60.00	65.00	66.67	65.00	64.16 ± 2.88
Ref_10	71.67	78.33	90.00	80.00	80.00 ± 7.57	68.33	68.33	81.67	61.67	70.00 ± 8.38
Ref_15	70.00	76.67	73.33	73.33	73.33 ± 2.72	55.00	66.67	71.67	55.67	62.25 ± 8.24
Ref_20	63.33	73.33	76.67	83.33	74.16 ± 8.33	66.67	78.33	78.33	75.00	74.58 ± 5.50
All	73.33	83.33	76.67	85.00	79.58 ± 5.51	56.67	83.33	85.00	71.67	74.16 ± 13.08

FD represents the feature dimension, and “All” refers to all dimensions, i.e., 60 × 484, *M* ± *SD* represents the mean and standard deviation of each feature selection dimension across the four neural networks. Bold and underlined values indicate the optimal results for features under different emotional conditions.

**Figures 22** and **23** illustrate the channel selection outcomes for the MDD and HC groups, respectively. The black dashed lines indicate the minimum number of features required to achieve optimal classification accuracy in each group. Correspondingly, **Table 13** lists the top-ranked channels contributing to the classification of positive versus negative emotional states. In the MDD group, channels 9, 5, 3, 4, and 8 emerged as the most critical for emotion recognition. In contrast, the HC group relied on a broader set of channels, with the most influential being 9, 2, 6, 21, 5, 11, 15, 13, 20, and 12.

**Figure 22:**
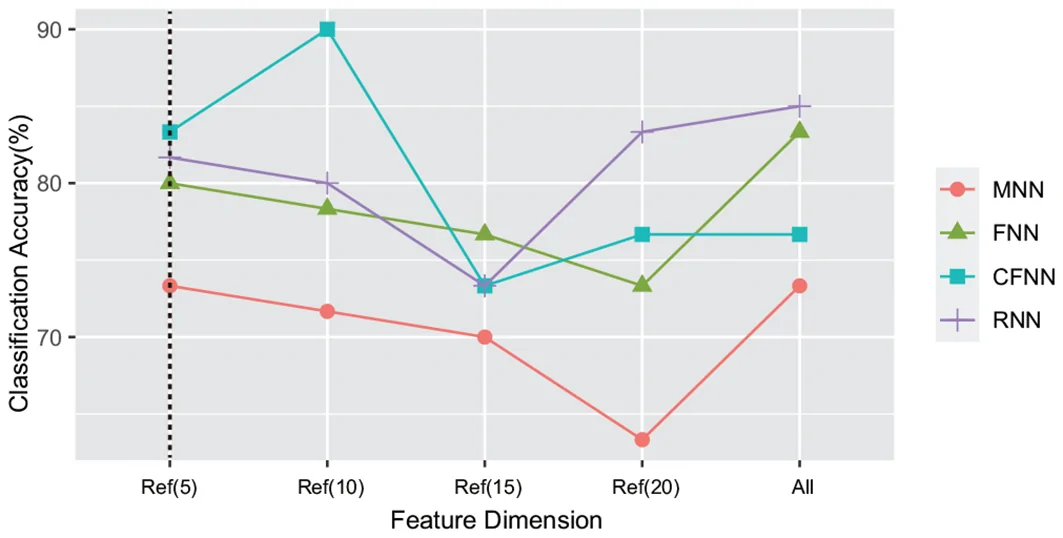
Relief F-based optimal feature selection results for the MDD group.

**Figure 23:**
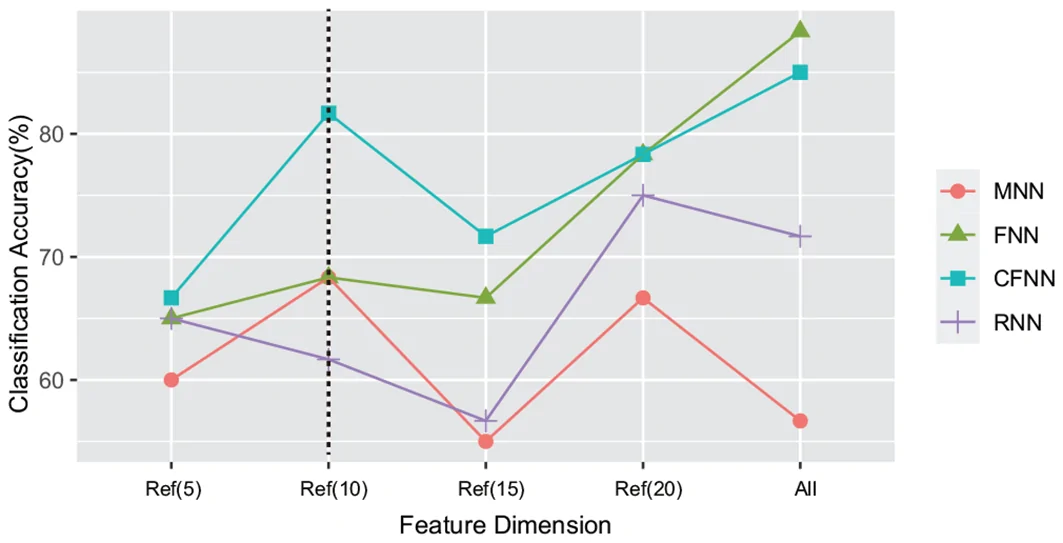
Relief F-based optimal feature selection results for the HC group.

**Table 13:** Ranking of channels with significant differences between MDD and HC groups across positive and negative emotions.

Group	Channel weight ranking
MDD	**CH9**, **CH15**, **CH3**, **CH4**, **CH8**, CH14, CH10
	CH5, CH11, CH17, CH22, CH12, CH16, CH18
	CH7, CH20, CH1, CH19, CH2, CH13, CH6, CH21
HC	**CH19**, **CH22**, **CH6**, **CH21**, **CH5**, **CH11**, **CH15**
	**CH13**, **CH20**, **CH12**, CH14, CH1, CH2, CH10
	CH17, CH18,CH16, CH4, CH7, CH8, CH9, CH3

Bold values indicate the optimal channel(s) for each emotional condition.

## 6 Conclusion

Numerous studies have demonstrated that MDD is associated with abnormal hemodynamic activity in multiple regions of the prefrontal cortex (38, 39). During cognitive or emotional tasks, increased HbO and decreased HbR concentrations typically reflect cortical activation (40). Meta-analyses of regional cerebral blood flow (rCBF) in MDD have reported increased resting-state rCBF in areas such as the right caudate, left insula, right posterior cingulate cortex, right hippocampus, and right precuneus, alongside reduced rCBF in the left inferior frontal gyrus, left anterior cingulate cortex, and left middle frontal gyrus—suggesting underlying metabolic dysfunction.

Building upon this evidence, the present study examined group-level differences in HbO concentration, prefrontal activation patterns based on GLM analysis, and β value–based classification performance during emotion recognition tasks in MDD patients versus healthy controls. The main findings are summarized as follows:

The MDD and HC groups demonstrated distinct patterns of HbO concentration in response to emotional stimulation. At the channel level, no significant differences were observed between groups under the happy and neutral conditions. However, under the fearful condition, significant group differences emerged in channels CH3 and CH6. Further comparison between positive and negative emotional states revealed widespread channel-level differences in the MDD group, whereas the HC group showed no significant distinctions.

Given the limitations of relying solely on hemodynamic concentration, we further examined group differences from the perspective of cortical activation. By mapping channel-level hemodynamic differences onto anatomically defined cortical regions, we found that the MDD group exhibited stronger and more spatially focused activation under the fearful condition compared to the HC group. Notably, significant activation differences were localized to the left dorsolateral superior frontal gyrus (SFGdor.L) under both positive and negative emotional conditions. In contrast, no region-specific activation differences were observed under the neutral condition. Within-group comparisons revealed consistent regional activation differences across emotional conditions in the MDD group, whereas no such differences were detected in the HC group. These findings underscore the altered emotional processing in MDD and highlight the need for more advanced analytical approaches—such as connectivity or network-level methods—when investigating emotion-related brain dynamics in healthy individuals.

Building on the observed differences in both channel-level and region-level features, classification experiments were conducted using *β* values under three emotional conditions to distinguish MDD patients from healthy controls, as well as to classify emotional valence within each group. Four BP neural network models achieved peak classification accuracies of 91.66%, 90.00%, and 93.33% under the happy, neutral, and fearful conditions, respectively. For within-group emotion recognition tasks, accuracies of 85.00% (MDD) and 88.33% (HC) were obtained. To optimize the balance between feature dimensionality and model performance, the ReliefF feature selection algorithm was applied, enabling the identification of the most informative channel subsets for classification.

In conclusion, this study identified both cortical activation and emotion-related hemodynamic differences between individuals with MDD and HC, providing empirical support for the hypothesis of disrupted hemodynamic regulation in MDD. These findings enhance our understanding of MDD-related neural dysfunction and highlight the potential of activation-based biomarkers for clinical assessment and emotion-informed diagnostic approaches.

## Data Availability

The raw data supporting the conclusions of this article will be made available by the authors, without undue reservation.
